# *In silico* investigation of riboswitches in fungi: structural and dynamical insights into TPP riboswitches in *Aspergillus oryzae*

**DOI:** 10.1080/15476286.2021.2015174

**Published:** 2022-01-06

**Authors:** Valdemir Vargas-Junior, Deborah Antunes, Ana Carolina Guimarães, Ernesto Caffarena

**Affiliations:** a Computational Biophysics and Molecular Modeling Group, Scientific Computing Programme (Procc - Fiocruz), Rio de Janeiro, Brazil; b Laboratory of Functional Genomics and Bioinformatics, Oswaldo Cruz Institute (Ioc - Fiocruz), Rio de Janeiro, Brazil

**Keywords:** TPP riboswitch, *aspergillus oryzae*, RNA, molecular dynamics, molecular modelling, ligand binding mechanism

## Abstract

Riboswitches are RNA sensors affecting post-transcriptional processes through their ability to bind to small molecules. Thiamine pyrophosphate (TPP) riboswitch plays a crucial role in regulating genes involved in synthesizing or transporting thiamine and phosphorylated derivatives in bacteria, archaea, plants, and fungi. Although TPP riboswitch is reasonably well known in bacteria, there is a gap in the knowledge of the fungal TPP riboswitches structure and dynamics, involving mainly sequence variation and TPP interaction with the aptamers. On the other hand, the increase of fungal infections and antifungal resistance raises the need for new antifungal therapies. In this work, we used computational approaches to build three-dimensional models for the three TPP riboswitches identified in *Aspergillus oryzae*, in which we studied their structure, dynamics, and binding free energy change (ΔG_bind_) with TPP. Interaction patterns between the TPP and the surrounding nucleotides were conserved among the three models, evidencing high structural conservation. Furthermore, we show that the TPP riboswitch from the *A. oryzae* NMT1 gene behaves similarly to the *E. coli* thiA gene concerning the ΔG_bind_. In contrast, mutations in the fungal TPP riboswitches from THI4 and the nucleoside transporter genes led to structural differences, affecting the binding-site volume, hydrogen bond occupancy, and ΔG_bind_. Besides, the number of water molecules surrounding TPP influenced the ΔG_bind_ considerably. Notably, our ΔG_bind_ estimation agreed with previous experimental data, reinforcing the relationship between sequence conservation and TPP interaction.

## Introduction

Riboswitches are cis-regulatory elements found in untranslated regions of the messenger RNA (mRNA) [1,2]. They act in the recognition and post-transcriptional regulation of specific metabolites, controlling the expression of genes related to their respective ligands’ synthesis and transport [1,3,4]. A riboswitch recognizes a particular metabolite and is responsible for gene regulation [5,6]. In addition, most of the riboswitches work by negative feedback, and their respective metabolites are sometimes essential for the organism’s maintenance [3,6–8].

Their mechanism of action is grounded on two interchangeable thermodynamically stable conformations, varying according to the presence or the absence of a specific ligand [9]. Although all riboswitches share their general mechanisms, distinct procedures are observed for the prokaryotic and eukaryotic organisms. In the first, riboswitches regulate transcription or translation processes [6,10,11], while in eukaryotes, regulation is carried out by alternative splicing [12,13].

Currently, over 40 riboswitches have been identified in bacteria, although the thiamine pyrophosphate (TPP) riboswitch stands out as the only class identified so far for eukaryotic organisms [5,6,13,14]. The TPP riboswitch recognizes the metabolite and regulates biosynthesis or transport of TPP (or its precursors) in a wide variety of organisms distributed among the three domains of life [5,10,14]. The metabolite TPP is a bioactive derivative of thiamine, composed of two moieties synthesized independently: thiazole (THZ) and pyrimidine (HMP) [13,15]. It acts as a cofactor in critical metabolic processes for obtaining energy, such as oxidative decarboxylation of pyruvate to get acetyl-coenzyme A, and in the pentose phosphate pathway, through the transketolase enzyme, for ribose synthesis [16–19].

**Figure 1.**
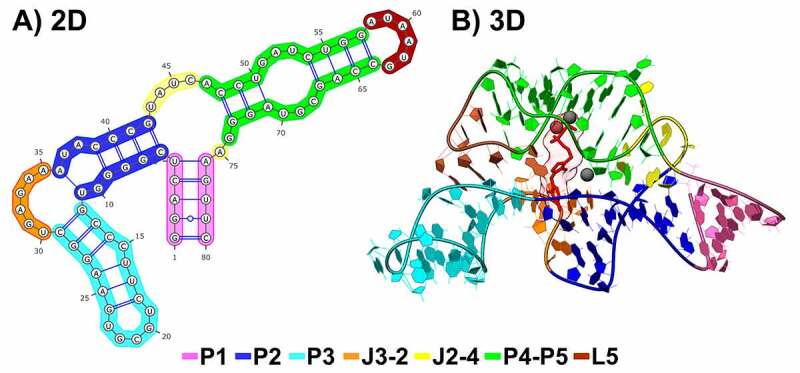
Structure of the TPP riboswitch aptamer from *Escherichia coli*, extracted from PDB ID: 2GDI. A) Secondary structure. B) Three-dimensional structure. Each aptamer region is distinguished by different colours, following the legend at the bottom of the image. The three-dimensional structure is bonded to TPP (shown in red) and three magnesium ions (dark grey spheres) in the binding site.

In general, a riboswitch is composed of an aptamer domain and an expression platform. Although the TPP riboswitch exists in phylogenetically distant organisms, its aptamer domain is sequentially and structurally conserved in all species, while its expression platform can vary [5,6,20]. The TPP riboswitch aptamer domain comprises different motifs, including five helices, two junctions between the helical motifs, and two loop regions (Figure 1(a)). The P1 helix connects the aptamer domain to the expression platform, while P2 and P3 interact mainly with the pyrimidine ring and stems P4 and P5 with the pyrophosphate group of TPP (Figure 1(b)). Depending on the organism, divergences among species are found mainly in the P3 region, varying in sequence, extension, and base-pairing content [5,21]. In eukaryotic organisms, the P3 region is commonly more extensive than in prokaryotes. Besides, bacteria and archaea commonly show a ramification of the P3 stem, known as P3a, not found yet in eukaryotes [21].

Several studies have investigated the biotechnological and therapeutic potential of bacterial TPP riboswitches [14,21,22]. However, fungal TPP riboswitches are still poorly explored in terms of dynamics and ligand binding behaviour. In contrast, the number of fungal infections in human beings has grown nearly 200% over the last few decades [23]. For instance, aspergillosis, a disease caused by species from *Aspergillus* genus, is believed to affect 300 million people worldwide, leading to nearly 1.6 million deaths, and has been reported as a secondary infection after covid-19 [23–25]. Moreover, fungal resistance is becoming a rising issue, mainly because of the few treatment options currently available, which makes the development of new antifungal compounds an emergency subject [26–29]. In this line, since bacterial TPP riboswitches have been pointed out as drug targets [14,30], understanding how fungal TPP riboswitches interact with their metabolite would help characterize ligand binding particularities in different structures and shed light on possible therapeutic and biotechnological approaches addressing medical fungi.

In this context, *Aspergillus oryzae* is a species ordinarily used as an experimental model among filamentous fungi, for which a validated TPP riboswitch secondary structure is available [31,32]. The existence of experimental data about a TPP riboswitch secondary structure is helpful to increase the accuracy of predicting its three-dimensional shape. Thus, it turns *A. oryzae* into an excellent subject to structural and dynamical investigation [20,31]. Moreover, earlier studies have identified that TPP riboswitches regulate at least three genes in *A. oryzae* [12]. The NMT1 and the THI4 genes account for the biosynthesis of TPP precursors HMP and THZ, respectively, while a third TPP riboswitch gene is believed to produce a protein that acts as a nucleoside transporter [12,13]. Therefore, from now on, we will refer to these TPP riboswitches from NMT1, THI4, and the nucleoside transporter as TPPsw^NMT1^, TPPsw^THI4,^ and TPPsw^NUC^, respectively.

In this work, we propose three-dimensional models for the aptamers of the TPP riboswitches from *A. oryzae*, in addition to the evaluation of the intermolecular interaction and affinity between fungal TPP riboswitches and their metabolite. We show that TPP riboswitches from *A. oryzae* and the *thiA* TPP riboswitch from *Escherichia coli* (TPPsw^EC^) behave similarly in structure and dynamics, while TPPsw^NUC^ and TPPsw^THI4^ acted differently concerning the binding free energy change (ΔG_bind_). As far as our concerns, this work stands as the first three-dimensional investigation of fungal TPP riboswitches.

## Results

### Comparative modelling

#### Bacterial and fungal TPP riboswitches share conserved sequences and structures

**Figure 2.**
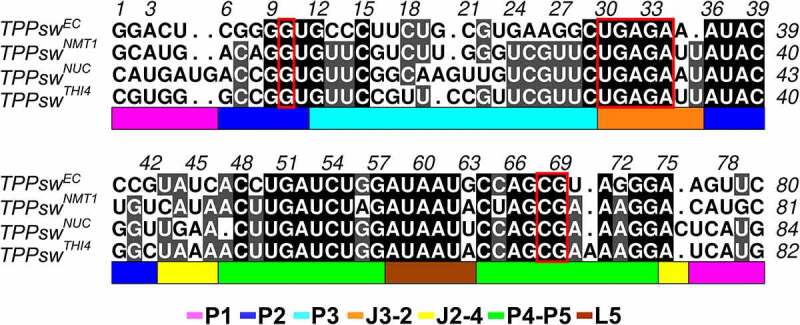
Multiple alignment between the *Escherichia coli* crystallographic structure (PDB ID: 2GDI) and *the Aspergillus oryzae* TPP riboswitches. The shading in black shows full identity between fragments, while the one in grey denotes partial identity. The absence of shading shows a lack of identity. Dots show the gaps between the strings. The coloured bars at the bottom of the alignment designate each nucleotide’s secondary structure in the aptamer, while the red rectangle signals TPP contacting nucleotides.

The multiple sequence alignment (MSA) between TPPsw^EC^ and the TPP riboswitches from *A. oryzae* revealed high conservation, with identities ranging from 57 to 62% (Figure 2). Overall, junction 3–2 (J3-2), loop 5 (L5), P4 and P5 stems appear the most conserved motifs, while P1 and P3 are the less conserved ones. In the L5 region, the first 5 out of the six composing nucleotides are also conserved. Thus, conformations assumed by the TPP riboswitch in its bound state are stabilized by contacts between L5 and the proximal portion of P3. In this context, although the P3 stem varies in content and extension in eukaryotic organisms [7], the first base pairing (G-C) of this motif is maintained in all sequences.

All the nucleotides interacting with TPP in TPPsw^EC^ are kept among the fungal sequences. However, TPPsw^THI4^ has an additional A (A72) in the P4 stem, located two nucleotides upstream G70, making contact with TPP. Similarly, TPPsw^NUC^ has an additional C (C79) in J2-4. Pairings C49-G72 from TPPsw^EC^ and G56-C65 from TPPsw^NMT1^ were replaced by AU pairings in the P4 and P5 stems, respectively.

We built, refined, and validated three models based on the MSA, one for each existing TPP riboswitch gene in *A. oryzae*. All the non-refined structures (including the template) presented clashes, deformed angles, and stretched bonds that were fixed after the refinement procedure, which improved their structure qualities and decreased the number of probably wrong sugar puckers and inadequate backbone conformations (supplementary material Table S1).

In agreement with the sequence conservation, an assessment performed in the SimTree server [33] revealed marked conservation among the secondary structures of the TPP riboswitches (supplementary material Table S2). However, minor differences were observed despite their similarity in sequence and secondary structures (supplementary material Figure S1). Those divergences concentrate on the P3 helix as a result of its lower conservation in comparison with other motifs.

**Figure 3.**
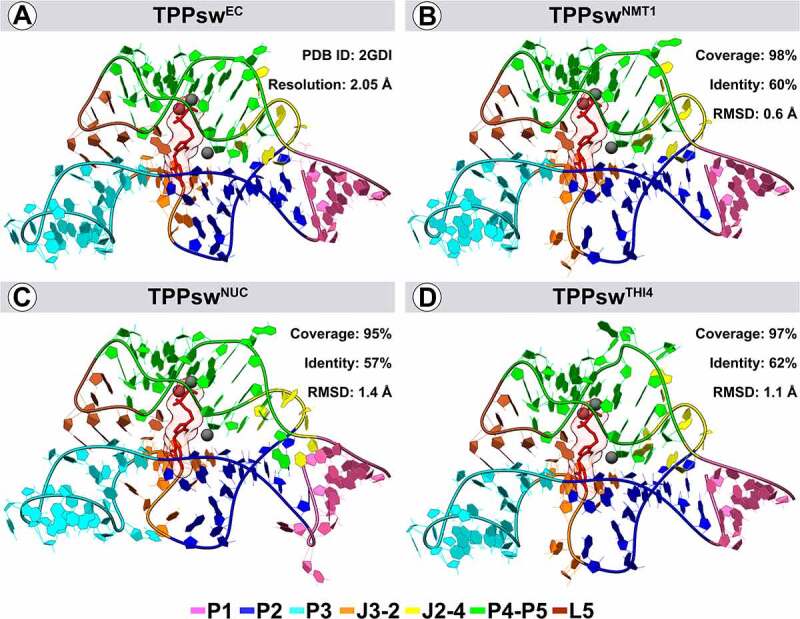
Three-dimensional structures of *Escherichia coli* (PDB ID: 2GDI) and *Aspergillus oryzae* models TPP riboswitches. The PDB ID of the template and resolution are given in the upper right corner of the upper left quadrant, while the RMSD, coverage degree, and identity between models and the template appear in the upper right corner of the other three. Colours show the position of each motif in the aptamer. The TPP ligand is shown in red, while the magnesium ions appear as spheres in dark grey. The three-dimensional structures were generated using the software UCSF Chimera version 1.14.

The TPPsw^EC^ and the fungal TPP riboswitches share similar 3D structures, although some differences can be observed. In general, the P3 helix portion (Figure 3) varied significantly, diverging between models and the template. TPPsw^NMT1^ showed the lowest RMSD value (0.6 Å) due to the most extensive coverage with the template (98.7%), while four additional nucleotides in the TPPsw^NUC^ sequence caused a slight displacement in P1/P2 and P4 stems compared with the template (Figure 2(c)).

**Figure 4.**
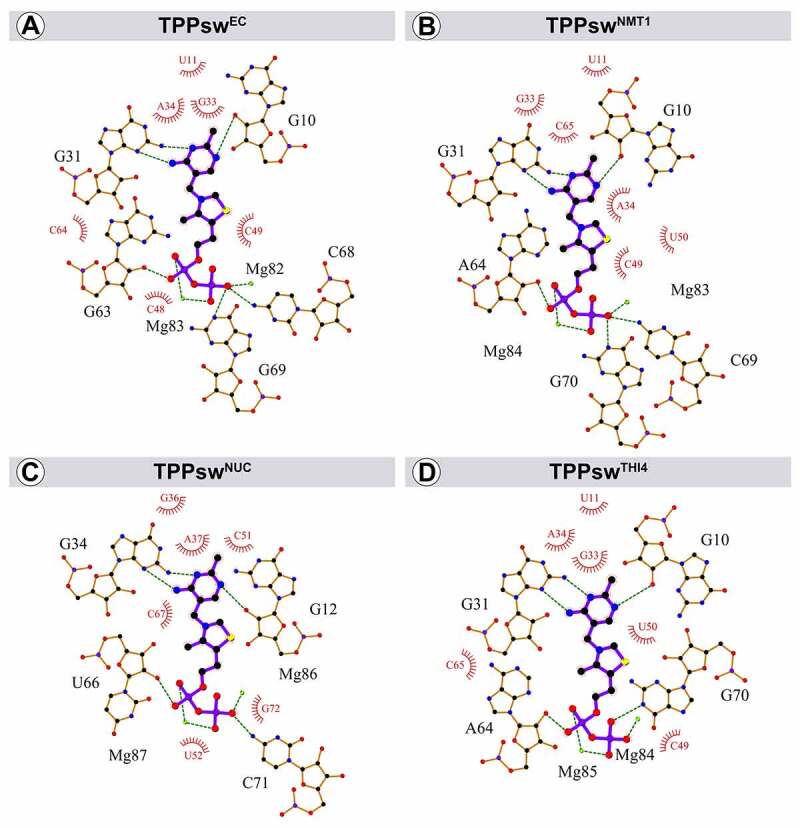
Interactions between TPP and TPP riboswitches. Dotted lines signal hydrogen bonds and interactions with magnesium ions. Green spheres refer to magnesium ions, red spheres to oxygen atoms, blue spheres to nitrogen atoms, and black spheres to carbon atoms. Hydrophobic contacts are depicted in red. The nucleotide numbering is shown next to each structure.

Interactions between the aptamers and the TPP ligand were also evaluated. In general, two magnesium ions help stabilize the TPP pyrophosphate group’s interaction with the binding site surroundings (Figure 4). Interactions between the TPP amino-pyrimidine ring and nucleotides G31 and G10 remained conserved in all structures, while G70 and C71 were absent in the TPPsw^NUC^ and TPPsw^THI4^ models, respectively, when compared with TPPsw^EC^.

All these interactions were previously reported [5], except the one between TPP and G63 (U66 in TPPsw^NUC^ and A64 in TPPsw^NMT1^ and TPPsw^THI4^), present in all the systems. Nevertheless, this nucleotide is not conserved at the sequence level, and consequently, it is not expected to keep a meaningful interaction with TPP over time.

In TPPsw^NUC^, G69(G71) is oriented towards the pyrophosphate group beyond the cut-off distance (3.3 Å) to characterize an atom-atom bond. An additional cytosine (C79) between P1 and P4 stems increases the distance between G69(G71) and TPP. Similarly, in TPPsw^THI4^, the hydrogen bond with TPP is lost due to the inclusion of A71 in the sequence. This nucleotide was found immediately upstream from G69(G70), increasing the distance between the adjacent C68(C69) and TPP (Figures. 2 and 4).

It is worth mentioning that these missing interactions in the models may provoke a decrease in structural stability or affinity loss. Therefore, we evaluated the dynamics of the TPP and the aptamer over time through molecular dynamics (MD) simulations to check these connections.

### Thermodynamical analysis

#### Affinity calculations suggest similar responses between TPP riboswitches

MD simulations allowed several assessments and shed light upon different aspects of the TPP riboswitch dynamical behaviour. Specifically, the ΔG_bind_ estimation between the TPP and the aptamer provides a perspective on the ligand recognition and the spontaneity of the interaction between the ligand and the receptor.

Overall, evaluations on all systems resulted in negative ΔG_bind_ values, indicating molecular association. The average ΔG_bind_ for the template (TPPsw^EC^) and TPPsw^NMT1^ was −10.2 ± 0.4 kcal/mol and −12.3 ± 0.7 kcal/mol, respectively (Table 1). The most negative ΔG_bind_ value was observed for the TPPsw^THI4^ (average: −16.5 ± 0.6 kcal/mol), estimated ~5 kcal/mol below its experimental value [14]. Regarding TPPsw^NUC^, the average ΔG_bind_ was around −5.1 ± 0.5 kcal/mol, being the model with less affinity with TPP.

**Table 1.** Binding free energy (ΔG_bind_)^a^ for all complexes of TPP riboswitches, calculated through MM/GBSA method

System	ΔE_vdw_	ΔG_esurf_	^b^Δ_ele+egb_	^c^ΔH	-TΔS	ΔG_bind_	^d^ΔG_exp_
**TPPsw^EC^**	−13.1 ± 0.3	−4.2 ± 0.0	−26.7 ± 1.3	−44.1 ± 0.3	33.9 ± 0.6	−10.2 ± 0.4	−12.4
**TPPsw^NMT1^**	−14.9 ± 0.2	−4.6 ± 0.0	−24.0 ± 1.4	−43.8 ± 0.3	31.4 ± 1.0	−12.3 ± 0.7	**^___^**
**TPPsw^NUC^**	−14.1 ± 0.2	−4.4 ± 0.0	−18.4 ± 1.6	−36.9 ± 0.3	31.7 ± 0.7	− 5.1 ± 0.5	**^___^**
**TPPsw^THI4^**	−14.0 ± 0.2	−4.3 ± 0.0	−32.1 ± 2.1	−50.6 ± 0.5	33.4 ± 0.6	−16.5 ± 0.6	−11.3

*^a^*All values are given in kcal/mol. *^b^*Δ_ele+egb_ = ΔE_ele_ + ΔG_egb_. *^c^*ΔH = ΔE_vdw_ + ΔE_ele_ + ΔG_esurf_ + ΔG_egb_. The average error is following the symbol ‘±’. *^d^*Experimental values were obtained from references [26] and [14].

TPPsw^EC^ and TPPsw^NMT1^ simulations showed ΔG_bind_ values close to the experimental ΔG_bind_ value reported for TPPsw^ec^ [34] diverging in less than 0.5 kcal/mol [34], also reproducible among the MD replicates (supplementary material Table S3). The most significant difference in ΔG_bind_ was observed in TPPsw^THI4^, achieving ~5 kcal/mol. As MM/GBSA usually does not excel in accuracy when compared to other methods (*e.g*. ABF and TI among others) [35,36], the ability to reproduce experimental data accurately, in this case, suggests high quality and reliability of the model building, refinement, and simulation processes. Besides, deviations in ΔG_bind_ calculations did not overcome 1 kcal/mol, showing a precise estimation of these quantities.

The ΔG_bind_ is defined as the difference between the enthalpy change (ΔH) and the absolute temperature multiplied by the entropy change (-TΔS_bind_). In our simulations, the -TΔS term resulted in comparable values for all systems, so the divergences in ΔG_bind_ observed among the models were enthalpically driven. On the other hand, the discrepancies in ΔH were mainly due to the Δ_ele+egb_ term that considers the polar contributions and the solvation free energy.

**Figure 5.**
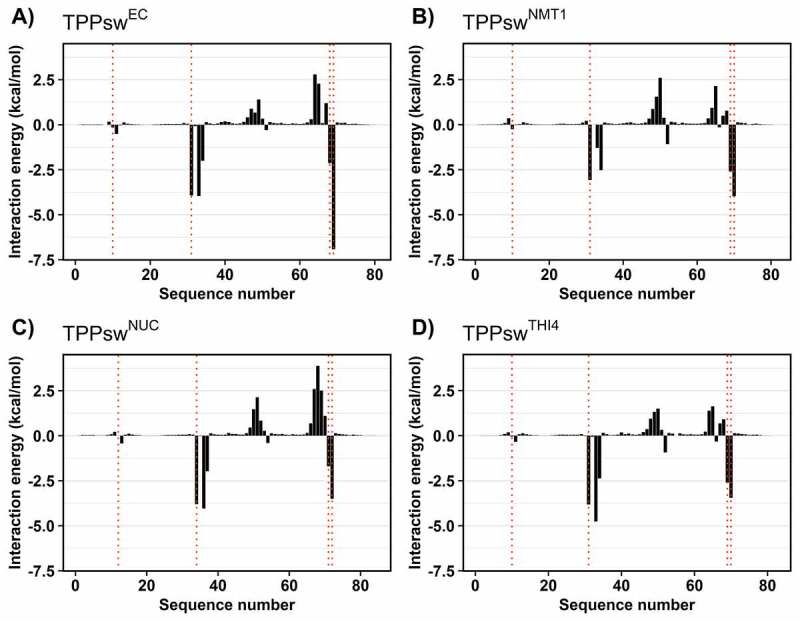
Decomposition of the binding free energy for the four simulated systems. Vertical lines dashed in red show those nucleotides interacting with the TPP (G10, G31, C68, and G69).

Each nucleotide’s contribution to the ΔG_bind_ was also assessed (Figure 5). Considering TPPsw^EC^ numbering, in general, the most negative values were observed for nucleotides G31, G33, A34 C68, and G69 (except for the G10) contacting TPP, and they show considerable contributions to the binding. In contrast, we also distinguished two groups of nucleotides with positive values in all systems. These nucleotides are located in the P4 and P5 stems near the TPP binding site, influencing the binding unfavourably. Noticeably, those positive contributions are smaller in TPPsw^THI4^, which resulted in the most negative ΔG_bind_ value. In contrast, for TPPsw^NUC^, those positive contributions were higher, achieving ~3.8 kcal/mol in C65(C68).

### Structural analysis

#### Water molecules in the TPP binding site in TPPsw^NUC^ interfere with aptamer-TPP interaction

**Figure 6.**
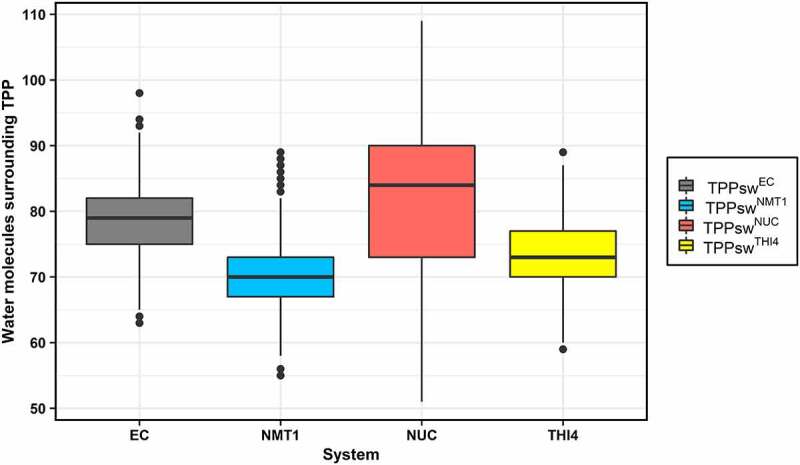
Boxplot of the number of water molecules within 3.5 Å of TPP. Each system comprises the last 50 ns of the three respective replicates. Systems are distinguished by different colours, according to the legend on the right side of the boxplot.

TPPsw^NUC^ and TPPsw^THI4^ show differences in sequence and secondary structure directly affecting the distance between TPP and specific regions of the aptamer and the solvent access surrounding TPP. In TPPsw^NUC^, the distance between P2 helix and TPP increases, allowing more water to interact with TPP, while in the other systems, the number of water molecules around TPP is lower (Fig. 6). Besides, the dispersion among TPPsw^NUC^ replicates was higher than other systems (82.12 ± 10.89), resulting in a wider interquartile range for TPPsw^NUC^.

A one-way analysis of variation (ANOVA) with a significance value of 0.05 was performed, resulting in a P-value of 2x10^−16^, denoting a discrepancy in the average number of water in the binding site between the four systems (Supplementary Table S4). In addition, a Tukey honest significance difference with a confidence level of 0.95 showed that the average number of waters also differs when comparing each possible pair of systems (Supplementary Table S5).

### Hydrogen bonds with TPP involve the identical nucleotides

**Figure 7.**
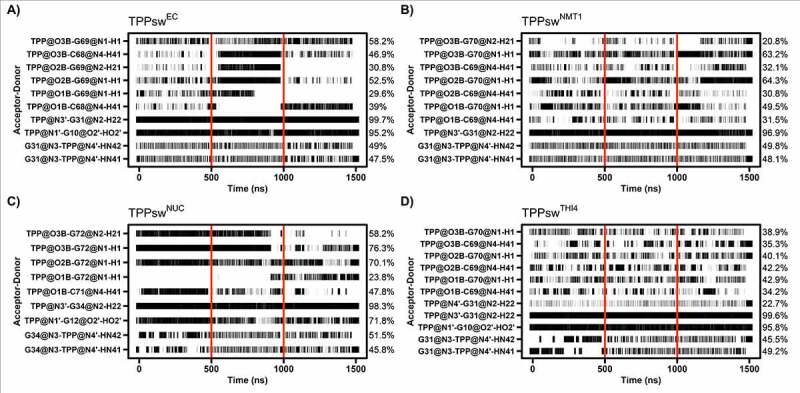
The hydrogen bond occupancy between the TPP atoms and the TPP riboswitch atoms is given in horizontal black bars, with its respective percentage on the right axis. The interacting pair is given on the left axis, while red vertical bars remark 500 ns long intervals.

Polar interactions are determinants for TPP binding. As both RNA and TPP are negatively charged units, the two structural Mg^+2^ ions are essential to balance the interactions and maintain the structure of the binding site. In general, those conserved Mg^+2^ ions help stabilize the pyrophosphate of TPP and help keep it oriented to C68 and G69. Before simulations, TPP interactions with C68 and G69 were absent in TPPsw^THI4^ and TPPsw^NUC^, respectively. Despite it, over the simulation time, both C68 and G69 formed hydrogen bonds (HB) with TPP in all systems (Figure 7).

Additionally, due to a conserved Mg^+2^ ion close to G69, this nucleotide sometimes hydrogen-bonds to TPP through two distinct atoms simultaneously. This split interaction competed with the TPP-C68 interaction, which resulted in lower HB occupancies. Therefore, although C69 interacted with TPP throughout all simulations, the occupancies were not high because of those interchangeable pairings (Figure 7).

HB with TPP mainly involved the nucleotides in conserved positions described in the interaction map (Figure 4), which agree with the lower interaction energies (Figure 5). The hydrogen bond between G63 and TPP (~5% occupancy) is not conserved, and the distance between them increased during simulation in all systems. The contact between the aptamer and the HMP region of TPP was kept especially through G31 (G31@N2–TPP@N4), which formed an HB that remained for more than 96% of the time in all systems, probably because of a stabilization provided by nucleotides G33 and A34 since they show pronounced attractive interaction energies (Figure 5). The exception for the interaction in HMP moiety is G10. Hydrogen bonding between G10 and TPP appears as a stable interaction in most systems, except TPPsw^NMT1^, occurring exclusively at the beginning of each simulation, remaining only 19.8% of the time. Noticeably, the energy decomposition showed values close to zero for G10 in all systems, indicating a less significant interaction with TPP.

### Structural stability of TPP and the TPP riboswitches in the aqueous medium

We have analysed the aptamer’s and ligand conformational behaviour and verified structural stability using RMSD and RMSF, taking their respective final configurations from the equilibration stage as a reference.

Regarding the aptamer’s stability, TPPsw^EC^ presented the slightest variation, fluctuating steadily around 2.5 Å throughout the simulation. Replicates also showed equivalent RMSD values with an associated error of ~0.2 Å. Likewise, RMSD values corresponding to the models kept steady, with TPPsw^NUC^ assuming the highest average RMSD (4.66 ± 0.63 Å), while TPPsw^NMT1^ and TPPsw^THI4^ oscillated around 3.5 with a deviation less than 0.4 Å (Supplementary material Figure S3). The TPP structure showed a continual behaviour in TPPsw^EC^ and TPPsw^THI4^, while TPPsw^NMT1^ and TPPsw^NUC^ revealed higher RMSD oscillations (Supplementary material Figure S2).

Although the observed RMSD values refer to the aptamer as a whole, the different structural regions contributed differently to the total average RMSD (Table 2). In general, higher RMSD values were frequent in the P1 and P3 regions, while motifs contacting TPP showed higher structural stability (Supplementary Figures S4 to S10).

**Table 2.** Average Root-mean-square deviation (RMSD) during 500ns of simulation

System	TPPsw^EC^	TPPsw^NMT1^	TPPsw^NUC^	TPPsw^THI4^
**Whole aptamer**	2.52 ± 0.33	3.46 ± 0.41	4.33 ± 0.24	3.44 ± 0.32
**P1**	1.62 ± 0.34	1.30 ± 0.21	3.74 ± 0.57	1.60 ± 0.73
**P2**	2.07 ± 0.69	2.21 ± 0.33	3.08 ± 0.47	2.25 ± 0.21
**P3-L3**	1.77 ± 0.34	3.16 ± 0.71	2.91 ± 0.53	2.61 ± 0.40
**P4-P5**	1.04 ± 0.16	1.32 ± 0.13	1.73 ± 0.28	3.24 ± 0.27
**J3-2**	1.17 ± 0.19	2.82 ± 0.52	2.88 ± 0.42	2.50 ± 0.40
**J2-4**	2.55 ± 0.21	2.97 ± 0.21	4.40 ± 0.84	3.09 ± 0.48
**L5**	1.51 ± 0.59	1.95 ± 0.34	1.29 ± 0.28	1.18 ± 0.19
**TPP**	1.75 ± 0.23	1.34 ± 0.35	1.51 ± 0.32	1.61 ± 0.10

All values are given in Å. Standard deviations are shown after the symbol ‘±’.

**Figure 8.**
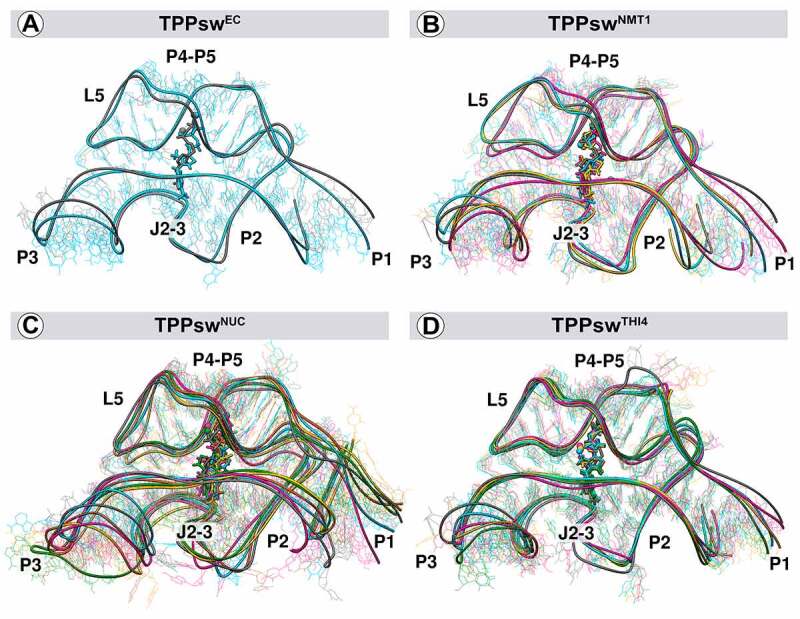
Representative structures of the cluster analysis. The largest clusters comprising nearly 90% of the conformations are shown for each system. The first cluster of TPPsw^EC^ represents 93.38% of the conformations. The first three clusters of TPPswNMT1 sum 93.34% of conformations. The sum of the first 5 clusters in TPPswNUC comprises 89.66% of conformations. The first 4 clusters in TPPswTHI4 represent 89.04% of the conformations. Grey stands for the reference structure, cyan for cluster 1, magenta for cluster 2, yellow for cluster 3, green for cluster 4, and brown for cluster 5.

We performed an RMSD-based clustering analysis (cut-off = 2.5 Å) to identify any relevant conformational changes in the structures over the simulations (Supplementary material, Figure S11, and Table S4). When considering the whole aptamer, convergence between replicates was less pronounced, particularly in TPPsw^NUC^ and TPPsw^THI4^ (Figure S11 A, C, E, and G), probably due to the high mobility of the P3 region. When repeated this analysis without considering the P3 stem, convergence among replicates became evident (Figure S11 B, D, F, and H). The most representative structures of each system present similar RMSD values, particularly those motifs contacting the TPP (Figure 8). Higher structural variations are concentrated in P3 and P1, although minor differences can be seen in J2-3 for all systems and the P4 stem of TPPsw^NUC^ and TPPsw^THI4^ (Figure 8(c,d)). In TPPsw^NUC^, nucleotides from the P4 stem are more distant from TPP in comparison with its initial structure (Figure 8(c)). In opposition, in TPPsw^THI4^, the P4 stem got closer to TPP after simulation (Figure. 8(d)).

**Figure 9.**
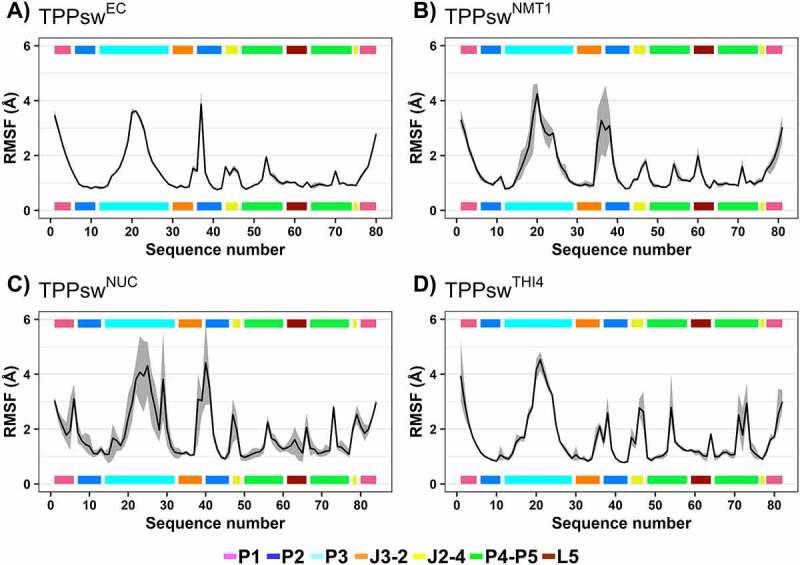
Root-mean-square fluctuation (RMSF). Fluctuation values are given in Å, per nucleotide. The black line shows average fluctuations between the models, while the grey shaded area shows the RMSF deviation between replicates. The coloured bars show the aptamer region, as stated in the legend.

The RMSF measures local flexibility and indicates how much a given nucleotide oscillated around its average position over time. All systems share similar fluctuation patterns (Figure 9), although TPPsw^EC^ showed the lowest variation among replicates’ fluctuation values, resulting in a negligible standard deviation. In contrast, TPPsw^NMT1^ and TPPsw^NUC^ showed higher disagreement between replicates, with the P2 and P3 helix being responsible for the most significant variations. All systems showed the lowest fluctuation associated with nucleotides surrounding TPP, indicating the high rigidity of these nucleotides. However, in TPPsw^NMT1^,TPPsw^NUC^ and TPPsw^THI4^, additional nucleotides in the aptamer provoked increased mobility of the motifs where they were inserted. As a matter of fact, all the nucleotides exclusive from the fungal TPP riboswitches showed high RMSF values, indicating greater flexibility of these points of the aptamers. For example, the U at the 3ʹ extremity of J2-3, present only in fungal TPP riboswitches, showed fluctuations ranging from 2 to 4 Å. Notably, in TPPsw^THI4^, a double peak of fluctuation was observed immediately after G70, with variations twice the values observed for these nucleotides in TPPsw^EC^ and TPPsw^NMT1^. Interestingly, the TPPsw^THI4^ replicates with the highest fluctuation near C69 and G70 also showed the most negative ΔG_bind_ values (Supplementary Table S3), suggesting an essential role of this region to the binding affinity of this region TPPsw^THI4^ with TPP. So, the addition of two nucleotides between P1 and P2 of TPPsw^NUC^ led to a fluctuation over 3 Å in this region. The same was observed for the additional C at the J2-4, which overcame 2.5 Å.

### The ΔG_bind_ is affected by the secondary structure change in TPPsw^NUC^ and TPPsw^THI4^

**Figure 10.**
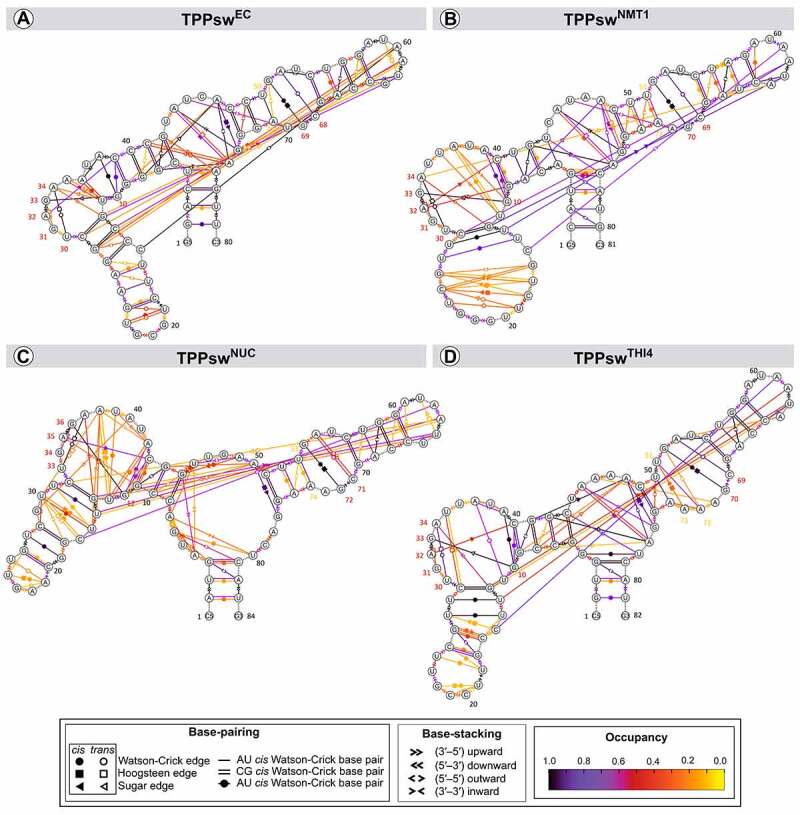
Dynamic secondary structure. Each trace describes a pairing, where the occupancy percentage is given in colours varying from yellow (zero) to black (one). The geometric forms in each line show the base-pairing type. Filled and hollow geometric forms stand for *cis* and *trans* interactions, respectively. Numbers coloured in red show nucleotides interacting with TPP.

TPP riboswitch bound conformation is maintained through non-Watson-Crick interactions between the L5 region and the proximal portion of the P3 helix [34,37,38] (Figure 10). Nucleotides C29 and A61 (in P3 and L5, respectively) form a Sugar-Hoogsteen interaction and constitute the primary base-pairing between these two regions in all systems. This interaction was weakened in TPPsw^NMT1^ because of the pairing between U13 (P3) and A61 (L5) that took around 20% of the simulation time.

A second meaningful interaction in TPPsw^EC^ is the Wobble pair formed between G28 and U(A)60. This interaction vanished in the models since the G nucleotide at position 60 was replaced by a U nucleotide. However, another pairing gained relevance, replacing the missing one in TPPsw^NUC^. The Wobble pair formed by G12 and U62 also plays a role in stabilizing the connection between the P3 and L5. This connection remained formed for ~ 60% of the time in TPPsw^NUC^, although it was less frequent in TPPsw^NMT1^ (~ 31%) and absent in TPPsw^THI4^.

The assessment of the secondary structure variation over time revealed that the core structures of the four aptamers were preserved in the simulations (Figure 10). Additional pairings were also observed all over the structures. An existing loop between P4 and P5 stems adopted a fully helical conformation in all the systems. Similarly, the P3 helix also adopted complete helical conformation, although less stable than P4 and P5, kept by several alternating weak pairings rather than stable single ones. This was observed even for TPPsw^NMT1^, which presented a P3 initial conformation fully unpaired.

Although the four systems share similar patterns of nucleotide pairing, the presence of an additional nucleotide in TPPsw^NUC^ (C79) interfered in pairings involving A78 (Figure 10(c)). In fact, in the other three systems, A78 (A75 in TPPsw^EC^) participates in several interactions with nucleotides from 3 motifs, while in TPPsw^NUC^, neither A78 nor C79 make pairings. Thus, the connectivity loss of A78 probably impaired the ΔG_bind_ in TPPsw^NUC^ since it separated TPP and P2, consequently allowing water molecules to access the binding site. Besides, the additional A in the TPPsw^THI4^ structure resulted in interchangeability between A72 and A73 for the pairing with U51. Thus, the expected pairing between U51 and A73 was confirmed, with ~30% occupancy, which alternated with A72–U51 pairing (~ 70% occupancy).

## Discussion

In bacteria, riboswitch regulation is relatively well understood. However, the scenario is different in fungi, where a lack of information exists [4,7,20,39]. Thus, despite the recent advances in fungal TPP riboswitch genomics, several gaps remain to be fulfilled, particularly regarding structure and dynamics. Here, our characterization of fungal TPP riboswitches brings important insights regarding ligand-binding behaviour.

Even among distantly related organisms, such as plants and bacteria, conservation between TPP riboswitch sequences and TPP regulation’s role remains a common aspect [5,6,14,34]. Antunes *et al*. (2018) have shown that TPPsw^EC^ and a TPP riboswitch from *Arabidopsis thaliana* share highly conserved sequences and structures with similar dynamical behaviour under similar conditions [5]. Here, we show that biosynthesis-related TPP riboswitches from *A. oryzae* are also closely related to *E. coli*. In our study, results concerning mobility and stability of the TPPsw^EC^ in *A. oryzae* were comparable to what was reported previously using TPPsw^EC^ [2018, 5]. Those findings support that TPP riboswitch is also highly conserved in a distantly related organism at structural and dynamical levels. In contrast, our ΔG_bind_ calculation through MM/GBSA method revealed distinct ligand-binding behaviours among systems.

The MM/GBSA method has as main advantages: relatively low computational cost, fast execution, and the possibility of calculating the absolute ΔG and decomposing the total free energy into sub-components, allowing the evaluation of the single contributions to the ΔG_bind_ [37,38,40]. On the other hand, MM/GBSA has some drawbacks, particularly the need for extensive sampling and its lack of accuracy in comparison with methods using explicit solvents [40]. Besides, the use of implicit solvent models in MM/GBSA, brings an inconsistency since the simulations (using explicit solvents) and the energy calculations do not use the same energy function [37,38,40,41]. Even though our ΔG_bind_ estimations using MM/GBSA reproduced experimental data [36] with a good agreement, while other literature attempts have achieved more discrepant ΔG_bind_ values for the same ligand, even using more sophisticated enhanced sampling calculation methods [8].

In general, both TPPsw^NMT1^ and TPPsw^THI4^ are similar to TPPsw^EC^ in terms of dynamical performances. However, this fact raises the question of why are TPPsw^NMT1^ and TPPsw^THI4^ different in terms of ΔG_bind_? Winkler and collaborators showed that the P3 extension does not impact gene controlling [7], while Sudarsan et al., 2005, pointed out that the additional A (A72) nucleotide in the P4 helix of TPPsw^THI4^ does not seem to interfere in TPP binding [14]. However, according to our findings, A72 seems to play a remarkable role in the ΔG_bind_ for TPPsw^THI4^. Specifically, our results suggest that the answer lies in the interaction between U51 and the nucleotides A72 and A73 in TPPsw^THI4^.

In TPPsw^THI4^, the interaction between U51 and A73 is conserved among the other sequences (*e.g*. this interaction is held by U50 and A71 in TPPsw^EC^). Notably, the alternative pairing U51-A72 seems to have a considerable impact on the local and neighbourhood flexibility, increasing the mobility of nucleotides around TPP. It is known that high mobility enhances the affinity and reduces the water’s surface tension, affecting the ΔH and, consequently, the ΔG_bind_ [42–4446^46^46^47^]. Thus, the approximation of C69 and G70 to those flexible nucleotides in TPPsw^THI4^ probably provokes an increment of the local kinetic energy, reducing the cohesion of the water molecules around TPP. Thus, although TPPsw^NMT1^ and TPPsw^THI4^ have a similar number of water molecules surrounding TPP, this higher flexibility in TPPsw^THI4^ could explain the more negative ΔH in TPPsw^THI4^ (Table 1).

In contrast, we noticed that TPPsw^NUC^ behaves differently in some aspects (*e.g*. binding free energy with TPP and overall stability). Although the differences in TPPsw^NUC^ might be due to a limitation inherent to the modelling process, our results and additional literature data lead to an alternative point of view. Structurally, the explanation for such a distinct behaviour can be due to three additional nucleotides (U6, G7, and C79) in the sequence than the other structures, leading to the distortions of the TPP binding site distance between TPP and its neighbouring nucleotides, achieving over 6 Å. When comparing TPPsw^NUC^ to other systems, the most peculiar difference is likely C79, which prevents the adjacent A78 from pairing with other nucleotides. This finding corroborates the fact that sequence conservation is essential for TPP riboswitch functioning.

Other interesting questions are: if sequence and structure conservation is essential to withstand TPP interaction, what is the consequence of having additional nucleotides at P1 and J2-4 of TPPsw^NUC^? The answer to this question might be the loss of gene expression regulation through TPP riboswitch. Donovan *et al*., 2018 described an ancient thiamine transporter in *Candida* species (an ortholog of *N. crassa* gene NCU01977) and a likely loss of riboswitch control in yeasts [45]. Also, Moldovan *et al*., 2018 described the TPPsw^NUC^ as a putative transporter and the thi9 gene [13]. Unfortunately, it is impossible to assure that those two putative transporters are functional TPP riboswitches due to the lack of experimental validation. Hence, a likely loss of function could explain why the sequence of TPPsw^NUC^ and thi9 are less conserved. Consequently, it could clarify why the ΔG_bind_ among TPPsw^NUC^ and TPP is lower than the observed for the biosynthesis-related TPP riboswitches. The findings from Moldovan *et al*., 2018 and Mukherjee *et al*., 2018 support this hypothesis since both showed that TPPsw^NUC^ and thi9 genes are considerably less abundant in fungi species than the other three fungal TPP riboswitches [12,13].

Finally, there is evidence of the potential of TPP riboswitch as a drug target for bacterial infections [14,30]. Despite the ΔG_bind_ differences among the four systems, we highlight that the overall dynamic behaviour of the *A. oryzae* TPP riboswitches is comparable to the one of TPPsw^EC^. In this context, our results suggest that TPP riboswitches might be drug targets for fungal infections as well, although further studies might be required for confirmation.

## Conclusion

We show that our findings indicate evident conservation in terms of structure and dynamics among bacterial and fungal riboswitches. In general, we show that the *Aspergillus oryzae* TPP riboswitches share conserved motifs with the bacterial TPPsw^EC^ and behave similarly in dynamics. Slight differences in sequence and structure might be enough to affect the binding free energy between TPP and the aptamer. We show that the biosynthesis-related TPP riboswitches TPPsw^NMT1^ interacts steadily with TPP, while the addition of three nucleotides in TPPsw^NUC^ weakened its interaction with TPP. In contrast, an alternative pairing, allowed by an additional adenine at the P4 helix of TPPsw^THI4^, enhanced the binding affinity between TPP and the aptamer. Our results strongly support that sequence conservation is essential to preserve TPP interaction with the aptamer and possibly maintain the TPP riboswitch function.

## Methods

We have chosen all the software utilized here based on previous literature references and considering their cost-benefit ratio to this work. Whenever possible, we preferred free software, given that it allows the reproducibility of this work to other groups.

### Target sequences

Sequences referring to the TPP-riboswitch aptamers in *A. oryzae* were obtained from the NCBI/Nucleotide database using the following codes and genomic coordinates (SC): ID: AKHY01000152.1. SC: 339156–338929 (TPPsw^NMT1^), ID: AKHY01000089.1. SC: 410308–410126 (TPPsw^NUC^) and ID: AKHY01000211.1. SC: 41779–41609 (TPPsw^THI4^). These genomic coordinates were obtained from Mukherjee *et al*. (2018).

### Comparative modelling and refinement of A. oryzae TPP riboswitches

We selected the three-dimensional structure of TPP riboswitch from *Escherichia coli* (PDB ID: 2GDI) [11] as a reference for constructing the *Aspergillus oryzae* TPP riboswitches models. We chose the template based on the crystal resolution (2.05 Å) and the availability of literature data regarding its structure and dynamics, which allows a reference parameter for simulation results [5,21,39]. The *E. coli* TPP riboswitch (TPPsw^EC^) comprises the aptamer domain bound to one thiamine pyrophosphate molecule and three magnesium ions. We refined the TPPsw^EC^ using the Phenix software tools to adjust angles and pockets to achieve the conformation standards used by the Molprobity webserver as reference [48]. First, the ‘*phenix.secondary_structure_restraints*’ tool was employed to create a constraint file, taking the secondary structure as a restriction. Then, the 3D-structure was submitted to the energy minimization process, using the ‘*phenix-geometry.minimization*’ tool with the Cornell *et al*. force field for nucleic acids, refined by Zgarbová *et a*l. [49,50].

A multiple alignment was performed between the sequence extracted from the template and the three TPP riboswitch sequences from *A. oryzae*, using the SARA-Coffee mode from the T-Coffee web server[51], and refined manually. The manual refinement uses a consensus secondary structure of each gene as a reference and matches the nucleotide positions to their respective positions in the consensus secondary structure [12].

As the P3 length does not interfere in gene regulation [7], we reduced its extension in the fungal sequences to match their length with the P3 helix from TPPsw^EC^. All other regions of the aptamers were kept unaltered [5,14]. This process was assisted by visual inspection of the secondary structures using the VARNA visualization software [52].

We built one model for each of the three TPP riboswitch sequences of *A. oryzae* by comparative modelling using the ModeRNA web server (http://genesilico.pl/modernaserver) by submitting the previously described alignment and the optimized template structure [53]. Thus, each model was structurally refined following the procedure described above. First, the secondary structures of the modelled PDBs were extracted using the ModeRNA web server [53] and inspected using VARNA [52]. Then, we mapped the ligand interaction using the software LigPlot+ [54]. Three-dimensional structures were generated using UCSF Chimera version 1.14 [55].

### Molecular dynamics simulations

The optimized models were submitted to Molecular Dynamics (MD) simulations using the Amber 20 software with the Cornell *et al*., nucleic acids force field, refined by Zgarbová *et a*l. [49,50,56,57]. We performed the parameterization of the ligand (TPP) using the Amber general force field (GAFF), with partial charges assigned with the ANTECHAMBER tool [58]. Molecular topologies of the complexes were created using the tLEaP tool [58].

The systems were immersed in triclinic boxes filled with TIP3P water molecules [59], and the minimum distance from the solute to the edge of the box was 12 Å. After hydration, magnesium and chloride ions were added to neutralize the systems. Electrostatic interactions were treated using the Particle Mesh Ewald (PME) method, with a cut-off of 10 Å, while the switching approach was applied to treat interactions between unbound atoms.

Geometry optimization and energy minimization were performed in cycles, in which the first thousand steps of each cycle were performed using the steepest descent algorithm and the following ones with the conjugate gradient algorithm. As a stop criterion, the maximum number of steps was set to 5,000. First, only the solvent and ions were left free. At the same time, the positions of the heavy atoms of the aptamer and the ligand were kept restrained using a harmonic potential of force constant of 5 kcal/(mol.Å^2^), which was gradually reduced in each optimization cycle until the last cycle was achieved and simulations performed entirely unrestrained.

Three replicates were generated by taking initial atomic velocities from different Maxwell-Boltzman distributions, corresponding to an initial temperature of 20 K, with the corresponding seeds provided by a pseudo-random number generator. The heating simulation process ran from 20 to 300 K, gradually increasing the temperature with a limit of 500,000 steps. The pressure was kept constant at 1 atm using the weak-coupling algorithm [60] and applying a harmonic potential to restrain atomic positions of the RNA heavy atoms and TPP with a force constant of 10 kcal/(mol.Å^2^), while ions and water molecules were left free. Initial velocities were taken from the Maxwell-Boltzman distribution at T =20 K. This process consisted of 500 ps using an integration time of 2 femtoseconds.

The equilibrium dynamics were performed during 4 ns, using position restrictions on the heavy atoms of the RNA and TPP, with force constants starting from 5 kcal/(mol.Å^2^) and gradually approaching zero at the end of the equilibrium phase. The temperature was kept constant at 300 K using the Langevin thermostat with a collision frequency γ = 2.0 ps^−1^.

Molecular dynamics were performed in the NPT statistical ensemble. The pressure was controlled using the Berendsen barostat [60] with an isotropic position scale. The dynamics production was divided into 10 stages, totalling 500 ns in each of the three replicates.

### Trajectory analysis

The MM/GBSA was performed in the Amber software using the last 50 ns of each simulation for the calculations [56], using the optimized Generalized Born (GB) model called OBC model defined by igb =2 [56]. For the energy decomposition, we applied a per-residue basis scheme, defined by idecomp =2 [56]. The MM/GBSA was applied to calculate the Gibbs free energy change ΔG. The equation reads:

ΔG=ΔH−TΔS

where *ΔH* denotes the enthalpy change, T is the absolute temperature, and ΔS stands for the entropy change. The ΔH was obtained according to the following equation: ΔH=ΔEvdw+ΔEele+ΔGesurf+ΔGegb.

where ΔEvdw stands for the Van der Waals interactions change, ΔEele accounts for the electrostatic energy change, ΔGesurf denotes the surface free energy change, and ΔGegb the generalized born free energy change.

Root-mean-square deviation (RMSD), root-mean-square fluctuation (RMSF), and the clustering analysis were performed with Gromacs [61]. Clustering analysis was performed using the GROMOS method with a cut-off of 2.5 Å. Calculation of the hydrogen bond occupations was performed using CPPTRAJ, using a geometric criterion. The cut-off distance and angle between the TPP and the aptamer were set to 3.5 Å and 120 degrees, respectively. The secondary structure variation was assessed using the Barnaba [62] and Mint Softwares [63], using the models and their respective trajectories as inputs.

## Supplementary Material

Supplemental Material
