# Supplementary material for: In silico investigation of riboswitches in fungi: structural and dynamical insights into TPP riboswitches in Aspergillus oryzae
Source: RNA Biol. 2022 Jan 6;19(1):90–103. doi: 10.1080/15476286.2021.2015174 (PMC8786325; doi:10.1080/15476286.2021.2015174)
Supplement: Supplemental Material [file KRNB_A_2015174_SM3166.zip › supplementary/Supplementary_material.docx]

**In silico investigation of riboswitches in fungi: structural and dynamical insights into TPP riboswitches in *Aspergillus oryzae***

**Valdemir Vargas-Junior^1^, Deborah Antunes^2*^, Ana Carolina Guimarães^2^, Ernesto Caffarena^1^**

^1^Computational Biophysics and Molecular Modeling Group, Scientific Computing Program (PROCC - FIOCRUZ), Rio de Janeiro, Brazil

^2^Laboratory of Functional Genomics and Bioinformatics, Oswaldo Cruz Institute (IOC - FIOCRUZ), Rio de Janeiro, Brazil

(*) Corresponding Author. E-mail address: deborah.santos@fiocruz.br (D. Antunes)

**SUPPLEMENTARY MATERIAL**

**THREE-DIMENTIONAL MODELS AVALIABILITY**

The three-dimensional structures used in this work are available in the link below:

<https://github.com/vargasjunior/TPP-riboswitch-models>

**FIGURES**

[**Figure S1.** Secondary structures of the 2GDI (template) and *Aspergillus oryzae* models. 3](#_Toc78464178)

[**Figure S2.** RMSD of the TPP over 500ns. 6](#_Toc78464179)

[**Figure S3.** Aptamers RMSD over 500ns. 7](#_Toc78464180)

[**Figure S4.** RMSD of P1 stem over 500ns. 8](#_Toc78464181)

[**Figure S5.** RMSD of P2 stem over 500ns. 8](#_Toc78464182)

[**Figure S6.** RMSD of P3 stem over 500ns. 9](#_Toc78464183)

[**Figure S7.** RMSD of Junction 3-2 over 500ns 9](#_Toc78464184)

[**Figure S8**. RMSD of Junction 2-4 over 500ns 10](#_Toc78464185)

[**Figure S9.** RMSD of P4 and P5 stems over 500ns 10](#_Toc78464186)

[**Figure S10**. RMSD of Loop 5 over 500ns.. 11](#_Toc78464187)

[**Figure S11.** Clustering analysis comprising the last 50 ns of each replicate.. 12](#_Toc78464188)

**TABLES**

[**Table S1.** Molprobity validation comparison between unrefined and refined models. 2](#_Toc78464238)

[**Table S2.** Normalized score of similarity between RNA secondary structures. 3](#_Toc78464239)

[**Table S3.** Binding free energy change (ΔG_bind_)^a^ among TPP and TPP riboswitch aptamers, calculated through MM/GBSA method for all MD replicates. 4](#_Toc78464240)

[**Table S4**. One-way Anova of the number of waters within 3.5Å of TPP. 5](#_Toc78464241)

[**Table S5.** Tukey HSD of the number of waters within 3.5 Å of TPP. 5](#_Toc78464242)

[**Table S6.** RMSD between each representative structure and it respective initial structure. 13](#_Toc78464243)

**Table S1.** Molprobity validation comparison between unrefined and refined models.

|  |  |  | **NOT REFINED** | | **REFINED** | | **GOAL** |
| --- | --- | --- | --- | --- | --- | --- | --- |
| **TPPsw^EC^** | All-Atom | Clashscore, all atoms: | 1.94 | | 11.28 | | 66^th^ percentile |
|  | Nucleic Acid Geometry | Probably wrong sugar puckers | 4 | 5.00% | 1 | 1.25% | 0 |
|  |  | Bad backbone conformations: | 10 | 12.82% | 11 | 13.75% | 5% |
|  |  | Bad bonds: | 2/1895 | 0.11% | 0/1970 | 0.00% | 0% |
|  |  | Bad angles: | 4/2953 | 0.14% | 0/2977 | 0.00% | 1% |
| **TPPsw^NMT1^** | All-Atom | Clashscore, all atoms: | 21.65 | | 9.6 | | 75^th^ percentile |
|  | Nucleic Acid Geometry | Probably wrong sugar puckers | 2 | 1.23% | 1 | 1.23% | 0 |
|  |  | Bad backbone conformations: | 12 | 12.35% | 10 | 12.35% | 5% |
|  |  | Bad bonds: | 22/1928 | 1.14% | 0/1928 | 0.00% | 0% |
|  |  | Bad angles: | 7/3003 | 0.23% | 3/3003 | 0.00% | 1% |
| **TPPsw^NUC^** | All-Atom | Clashscore, all atoms: | 80.3 | | 10.76 | | 66^8h^ percentile |
|  | Nucleic Acid Geometry | Probably wrong sugar puckers | 2 | 2.38% | 1 | 1.19% | 0 |
|  |  | Bad backbone conformations: | 17 | 20.24% | 12 | 14.29% | 5% |
|  |  | Bad bonds: | 36/2006 | 1.79% | 0/1970 | 0.00% | 0% |
|  |  | Bad angles: | 27/3126 | 0.86% | 0/2977 | 0.10% | 1% |
| **TPPsw^THI4^** | All-Atom | Clashscore, all atoms: | 25.13 | | 10.66 | | 66^8h^ percentile |
|  | Nucleic Acid Geometry | Probably wrong sugar puckers | 2 | 2.44% | 1 | 2.44% | 0 |
|  |  | Bad backbone conformations: | 12 | 14.63% | 12 | 14.63% | 5% |
|  |  | Bad bonds: | 28/1952 | 1.43% | 0/1970 | 0.00% | 0% |
|  |  | Bad angles: | 13/3041 | 0.43% | 3/2977 | 0.10% | 1% |

Green indicates optimal values, yellow indicates acceptable values and red indicates inadequate values.


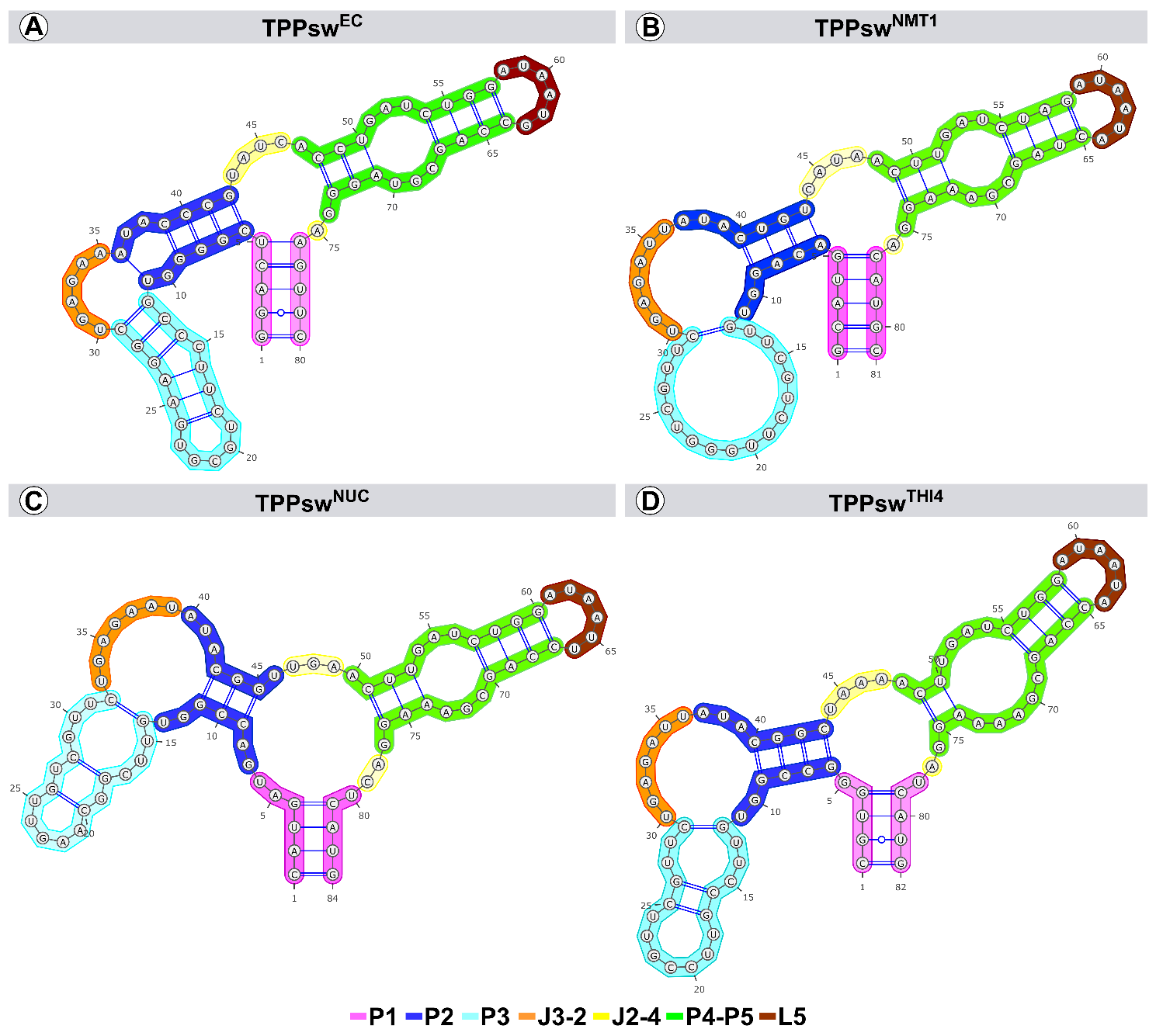


**Figure S1.** Secondary structures of the 2GDI (template) and *Aspergillus oryzae* models. Colors indicate the position of each motif in the aptamer

**Table S2.** Normalized score of similarity between RNA secondary structures.

| System | TPPsw^EC^ | TPPsw^NMT1^ | TPPsw^NUC^ | TPPsw^THI4^ |
| --- | --- | --- | --- | --- |
| TPPsw^EC^ | 1 | 0.8108 | 0.9030 | 0.8788 |
| TPPsw^NMT1^ | 0.8108 | 1 | 0.8184 | 0.8511 |
| TPPsw^NUC^ | 0.9030 | 0.8184 | 1 | 0.8687 |
| TPPsw^THI4^ | 0.8788 | 0.8511 | 0.8687 | 1 |

The normalization is on a scale from 0 to 1, where 1 indicates a perfect match and 0 no match, we found normalized scores of 0.81, 0.90, and 0.87 for the, respectively, taking the template as a reference.

**Table S3.** Binding free energy change (ΔG_bind_)^a^ among TPP and TPP riboswitch aptamers, calculated through MM/GBSA method for all MD replicates.

| **System** | | **Δe_vdw_** | **Δe_ele_** | **Δg_egb_** | **Δg_esurf_** | **^b^Δ_ele+egb_** | **^c^ΔH** | **-TΔS** | **ΔG_bind_** | **^e^ΔG_exp_** |
| --- | --- | --- | --- | --- | --- | --- | --- | --- | --- | --- |
| **TPPsw^EC^** | Rep1 | -13.69 ± 0.45 | 1955.78 ± 2.16 | -1981.21± 1.69 | -4.26 ± 0.009 | -25.43 ± 1.92 | -43.39 ± 0.45 | 30.56 ± 0.35 | -12.83 ± 0.40 | -12.48 |
|  | Rep2 | -9.99 ± 0.26 | 1900.72 ± 1.20 | -1933.90 ± 1.04 | -4.01 ± 0.007 | -33.17 ± 1.12 | -47.19 ± 0.25 | 38.52 ± 0.80 | -8.67 ± 0.52 |  |
|  | Rep3 | -15.91 ± 0.23 | 1880.23 ± 2.00 | -1901.79 ± 1.70 | -4.37 ± 0.007 | -21.56 ± 1.85 | -41.84 ± 0.33 | 32.68 ± 0.73 | -9.16 ± 0.56 |  |
|  | **Mean** | **-13.19 ± 0.31** | **1912.24 ± 1,78** | **-1938.96 ± 1.47** | **-4.21 ± 0.008** | **-26.72 ± 1.39** | **-44.14 ± 0.34** | **33.92 ± 0.62** | **-10.22 ± 0.49** |  |
| **TPPsw^NMT1^** | Rep1 | -13.14 ± 0.29 | 1949.74 ± 1.95 | -1975.34 ± 1.53 | -4.88 ± 0.005 | -25.60 ± 1.74 | -43.62 ± 0.41 | 30.05 ± 0.89 | -13.57 ± 0.69 | ___ |
|  | Rep2 | -12.59 ± 0.25 | 1918.05 ± 1.66 | -1945.02 ± 1.30 | -4.85 ± 0.005 | -26.97 ± 1.48 | -44.41 ±0 .43 | 31.02 ± 1.09 | -13.39 ± 0.82 |  |
|  | Rep3 | -19.13 ± 0.26 | 1981.33 ± 1.29 | -2000.82 ± 1.10 | -4.75 ± 0.007 | -19.48 ± 1.20 | -43.38 ± 0.35 | 33.16± 1.13 | -10.12 ± 0.74 |  |
|  | **Mean** | **-14.95 ± 0.26** | **1949.70 ± 1.63** | **-1973.72 ± 1.31** | **-4.63 ± 0.006** | **-24.01 ± 1.47** | **-43.80 ± 0.39** | **31.41 ± 1.03** | **-12.36 ± 0.75** |  |
| **TPPsw^NUC^** | Rep1 | -20.45 ± 0.24 | 2059.64 ± 2.28 | -2076.02 ± 2.11 | -4.89 ± 0.010 | -16.38 ± 2.19 | -41.73 ± 0.29 | 34.27± 0.45 | -7.46 ± 0.37 | ___ |
|  | Rep2 | -9.41 ± 0.24 | 2066.74 ± 1.27 | -2087.52 ± 1.08 | -4.22 ± 0.007 | -20.78 ± 1.17 | -34.42 ± 0.28 | 29.38 ± 0.78 | -5.03 ± 0.53 |  |
|  | Rep3 | -12.45 ± 0.23 | 2096.90 ± 1.59 | -2115.03 ± 1.29 | -4.18 ± 0.006 | -18.12 ± 1.44 | -34.76 ± 0.38 | 31.73 ± 1.09 | -3.03 ± 0.73 |  |
|  | **Mean** | **-14.10 ± 0.24** | **2074.42 ± 1.71** | **-2092.85 ± 1.49** | **-4.43 ± 0.007** | **-18.42 ± 1.6** | **-36.97 ± 0.31** | **31.79 ± 0.77** | **- 5.17 ± 0.54** |  |
| **TPPsw^THI4^** | Rep1 | -13.08 ± 0.24 | 2061.92 ± 2.31 | -2093.14 ± 1.84 | -4.27 ± 0.006 | -31.22 ± 2.07 | -48.58 ± 0.47 | 35.13 ± 0.78 | -13.45 ±0.64 | -11.32 |
|  | Rep2 | -16.17 ± 0.27 | 2071.29 ± 2.48 | -2102.93 ± 1.74 | -4.52 ± 0.005 | -31.64 ± 2.11 | -52.33 ± 0.71 | 33.50 ± 0.64 | -18.83 ± 0.67 |  |
|  | Rep3 | -13.04 ± 0.26 | 2098.60 ± 2.40 | -2132.23 ± 1.93 | -4.33 ± 0.007 | -33.63 ± 2.16 | -51.01 ± 0.49 | 33.58 ± 0.62 | -17.43 ±0.55 |  |
|  | **Mean** | **-14.09 ± 0.26** | **2077.27 ± 2.39** | **-2109.38 ± 1.83** | **-4.37 ± 0.006** | **-32.16 ± 2.11** | **-50.64 ± 0.55** | **33.45 ± 0.68** | **-16.57 ± 0.62** |  |

*^a^*All of the values are given in kcal/mol. *^b^*Δ_ele+egb_ = ΔE_ele_ + ΔG_egb._ *^c^*ΔH = ΔE_vdw_ + ΔE_ele_ + ΔG_esurf_ + ΔG_egb._ The average error is following the symbol "±". *^d^*Experimental values were obtained from references (36) and (8)

**Table S4**. One-way Anova of the number of waters within 3.5Å of TPP.

| SUMMARY |  |  |  |  |  |  |
| --- | --- | --- | --- | --- | --- | --- |
| System | Count | Sum | Average | Variance |  |  |
| TPPsw^EC^ | 1500 | 117662 | 78,44133 | 25,09262 |  |  |
| TPPsw^NMT1^ | 1500 | 105260 | 70,17333 | 23,52964 |  |  |
| TPPsw^NUC^ | 1500 | 123194 | 82,12933 | 118,8091 |  |  |
| TPPsw^THI4^ | 1500 | 110140 | 73,42667 | 24,86653 |  |  |
|  |  |  |  |  |  |  |
| ANOVA |  |  |  |  |  |  |
| Source | Sum sq | DF | Meam sq | F | P-value | Critical-F |
| Among groups | 126140,5 | 3 | 42046,82 | 874,6182 | 2x10^-16^ | 2,60639 |
| Residual | 288254,6 | 5996 | 48,07449 |  |  |  |
|  |  |  |  |  |  |  |
| Total | 414395,1 | 5999 |  |  |  |  |

**Table S5.** Tukey HSD of the number of waters within 3.5 Å of TPP.

| System | Difference | Lower | Upper | P adjusted |
| --- | --- | --- | --- | --- |
| TPPsw^EC^-TPPsw^NMT1^ | -8.268000 | -8.918606 | -7.617394 | 0 |
| TPPsw^EC^-TPPsw^NUC^ | 3.688000 | 3.037394 | 4.338606 | 0 |
| TPPsw^EC^-TPPsw^THI4^ | -5.014667 | -5.665272 | -4.364061 | 0 |
| TPPsw^NMT1^-TPPsw^NUC^ | 11.956000 | 11.305394 | 12.606606 | 0 |
| TPPsw^NMT1^-TPPsw^THI4^ | 3.253333 | 2.602728 | 3.903939 | 0 |
| TPPsw^NUC^-TPPsw^THI4^ | -8.702667 | -9.353272 | -8.052061 | 0 |

Each pair of systems is a matrix with 4 columns on which “Difference” gives the difference in the observed means, “Lower” gives the lower end point of the interval, “Upper” giving the upper end point and “P adjusted” giving the p-value after adjustment for the multiple comparisons.


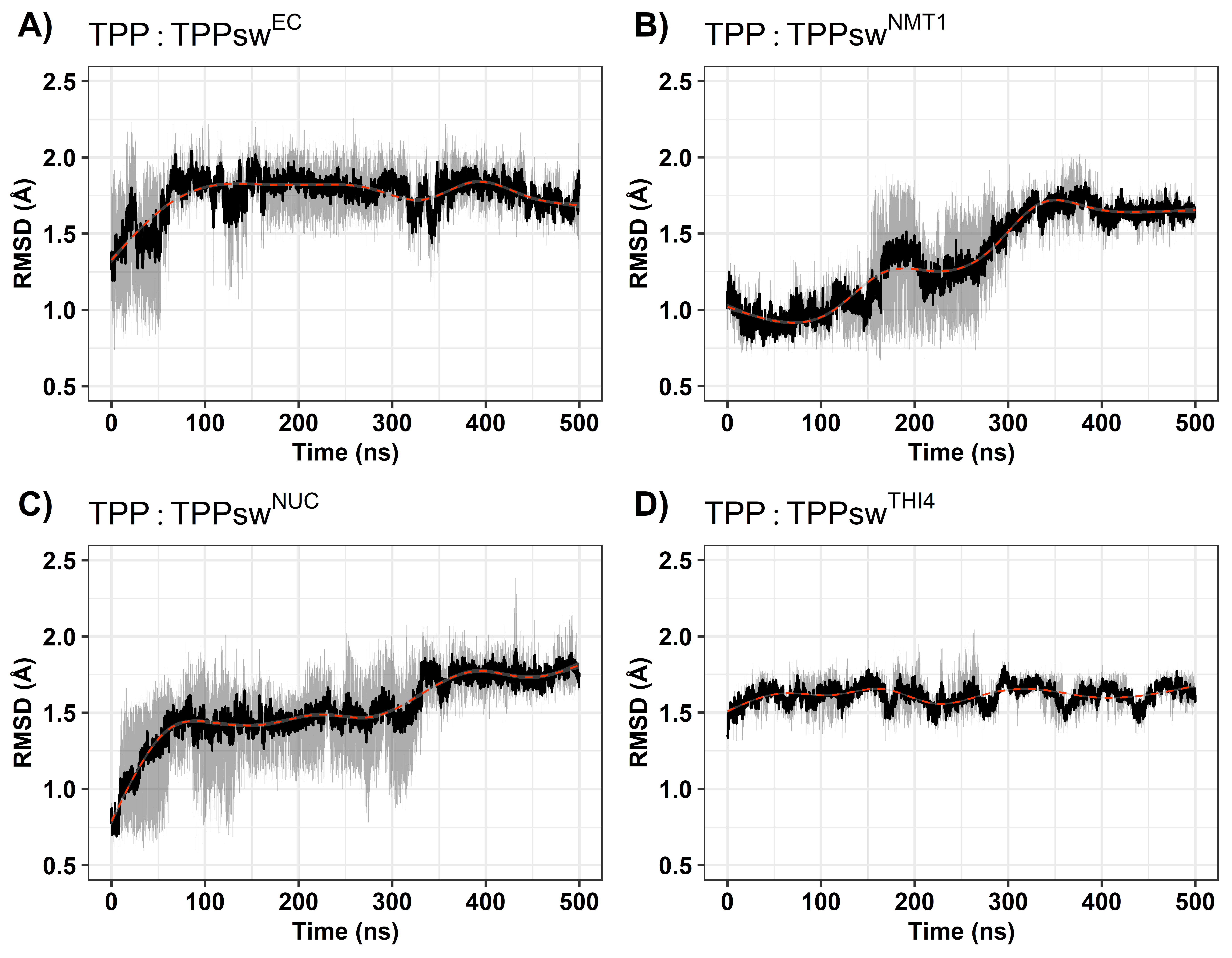


**Figure S2.** RMSD of the TPP over 500ns. The darker line indicates the average RMSD values of the three simulations. The gray area indicates the standard deviation between the values observed in each replicate, while the red dashed line indicates the trend of the values.


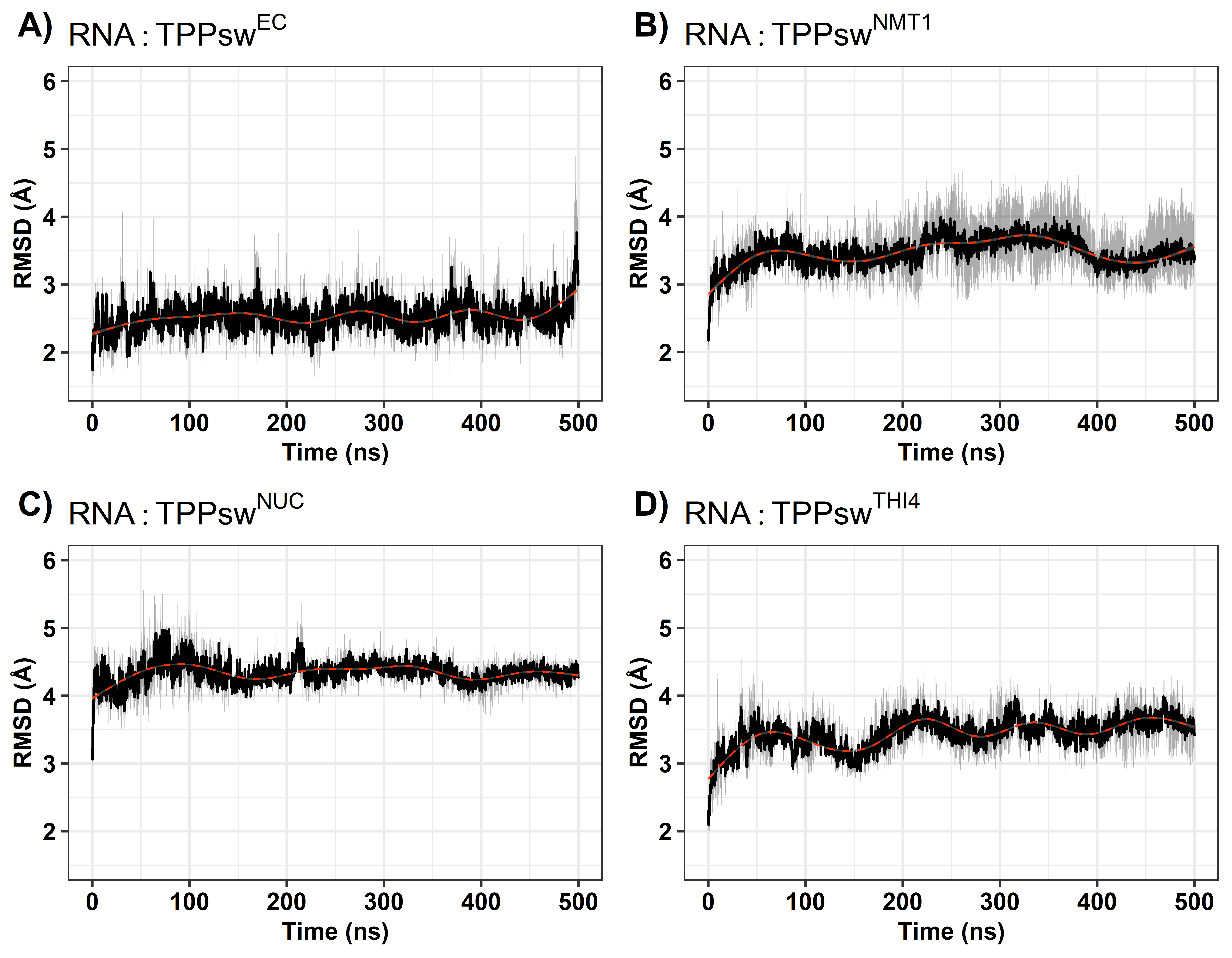


**Figure S3.** Aptamers RMSD over 500ns. The darker line indicates the average RMSD values of the three simulations. The gray area indicates the standard deviation between the values observed in each replicate, while the red dashed line indicates the trend of the values.


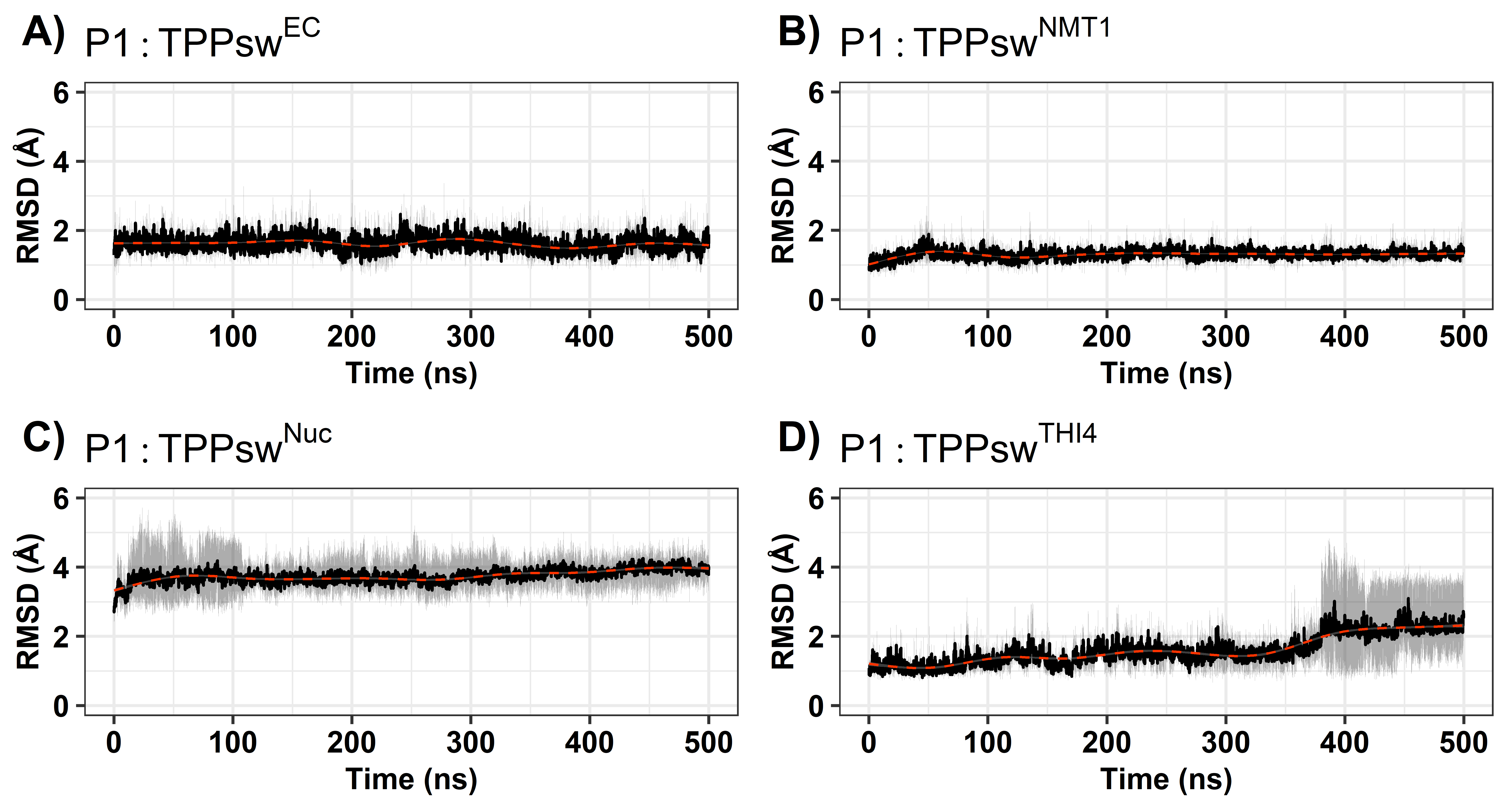


**Figure S4.** RMSD of P1 stem over 500ns. The darker line indicates the average RMSD values of the three simulations. The gray area indicates the standard deviation between the values observed in each replicate, while the red dashed line indicates the trend of the values.


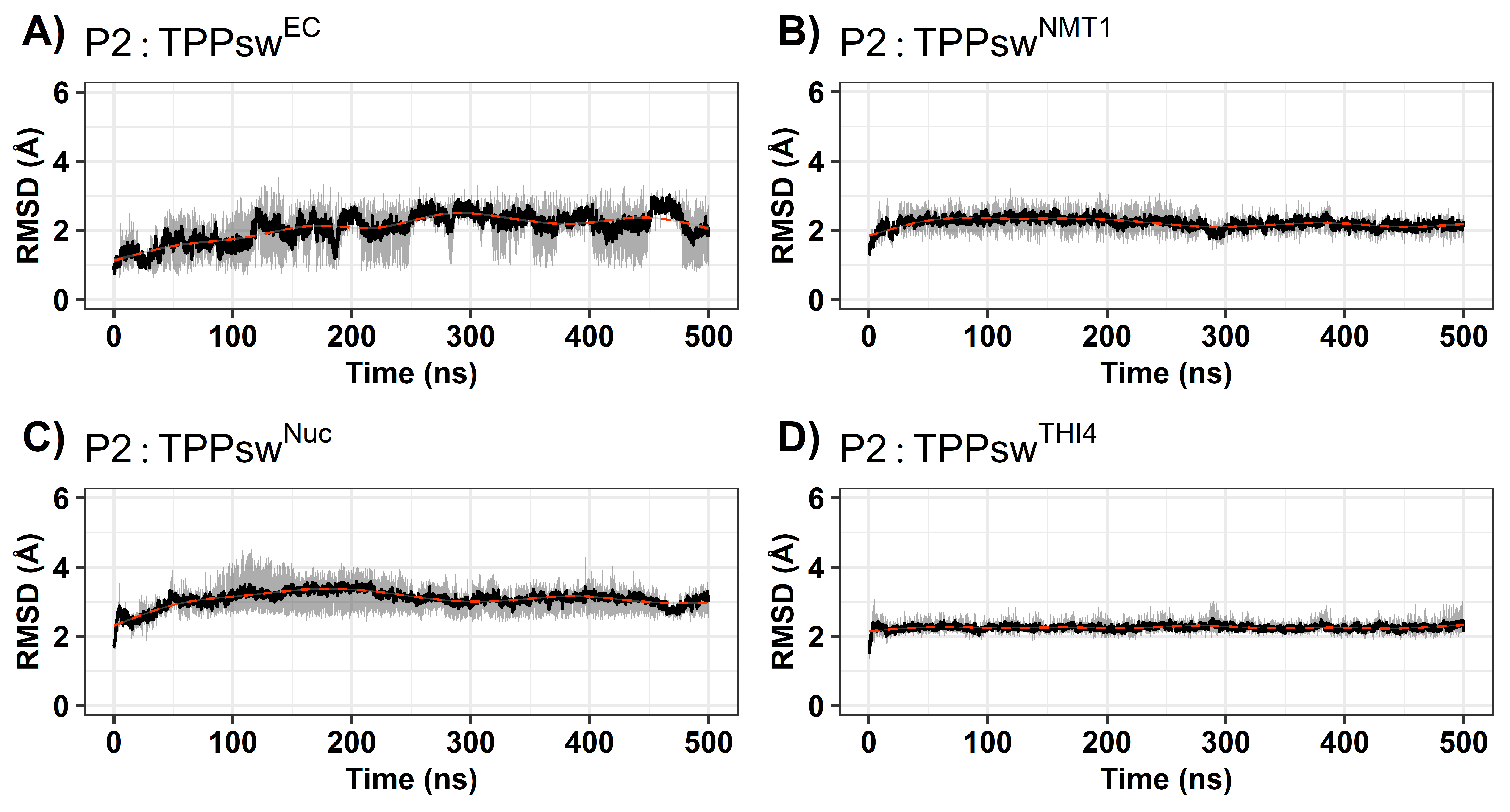


**Figure S5.** RMSD of P2 stem over 500ns. The darker line indicates the average RMSD values of the three simulations. The gray area indicates the standard deviation between the values observed in each replicate, while the red dashed line indicates the trend of the values.


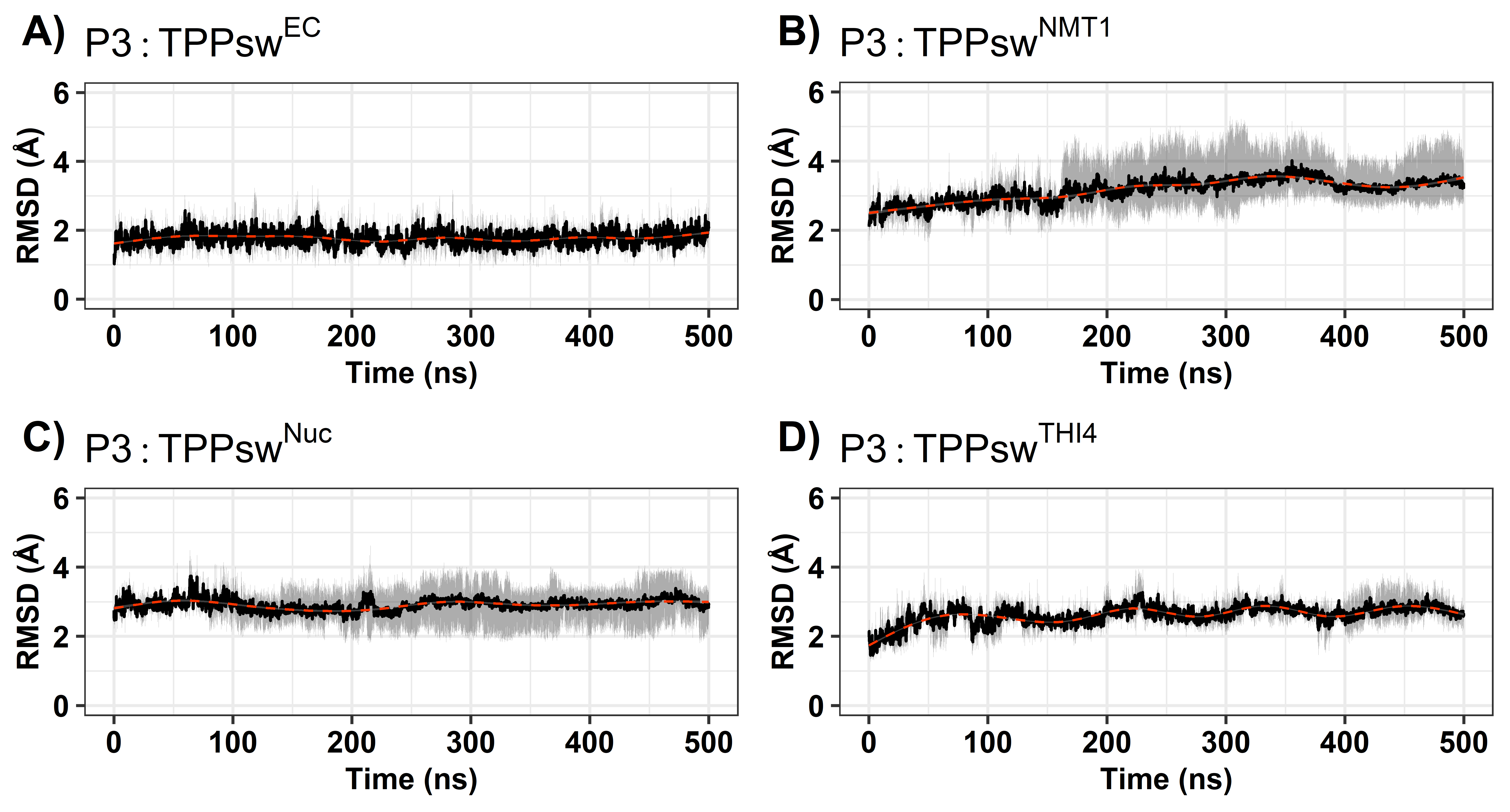


**Figure S6.** RMSD of P3 stem over 500ns. The darker line indicates the average RMSD values of the three simulations. The gray area indicates the standard deviation between the values observed in each replicate, while the red dashed line indicates the trend of the values.


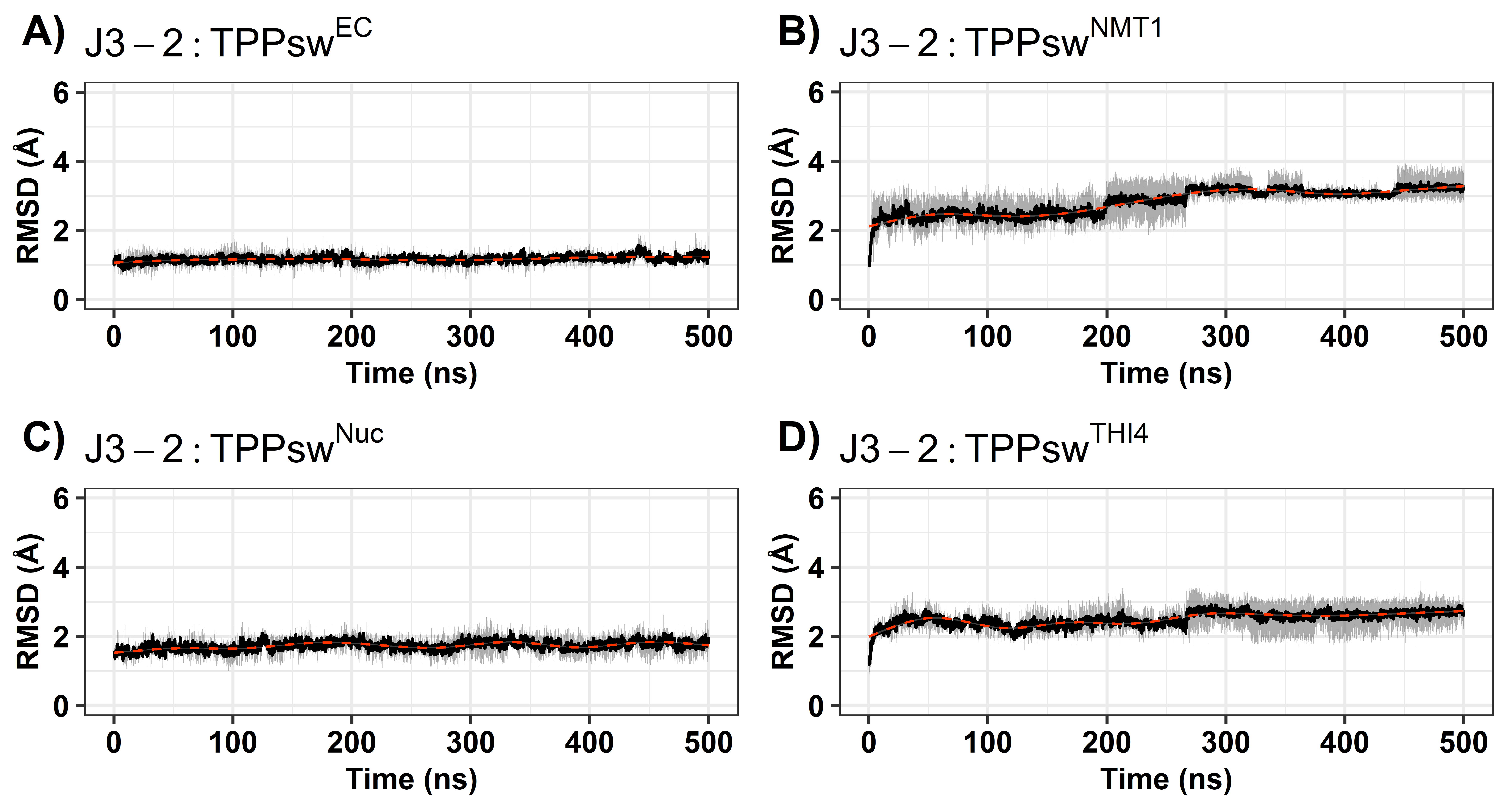


**Figure S7.** RMSD of Junction 3-2 over 500ns. The darker line indicates the average RMSD values of the three simulations. The gray area indicates the standard deviation between the values observed in each replicate, while the red dashed line indicates the trend of the values.


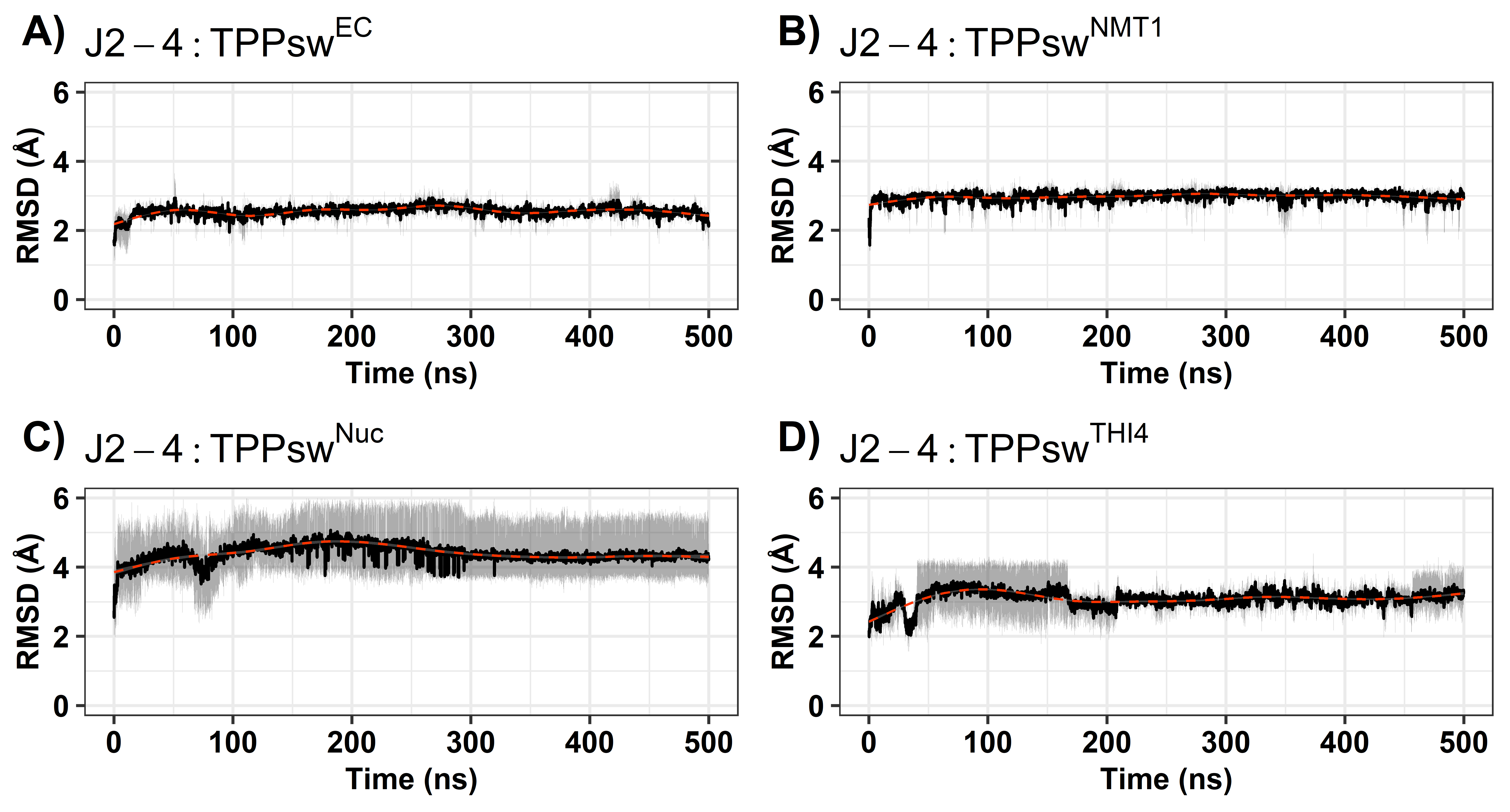


**Figure S8**. RMSD of Junction 2-4 over 500ns. The darker line indicates the average RMSD values of the three simulations. The gray area indicates the standard deviation between the values observed in each replicate, while the red dashed line indicates the trend of the values.


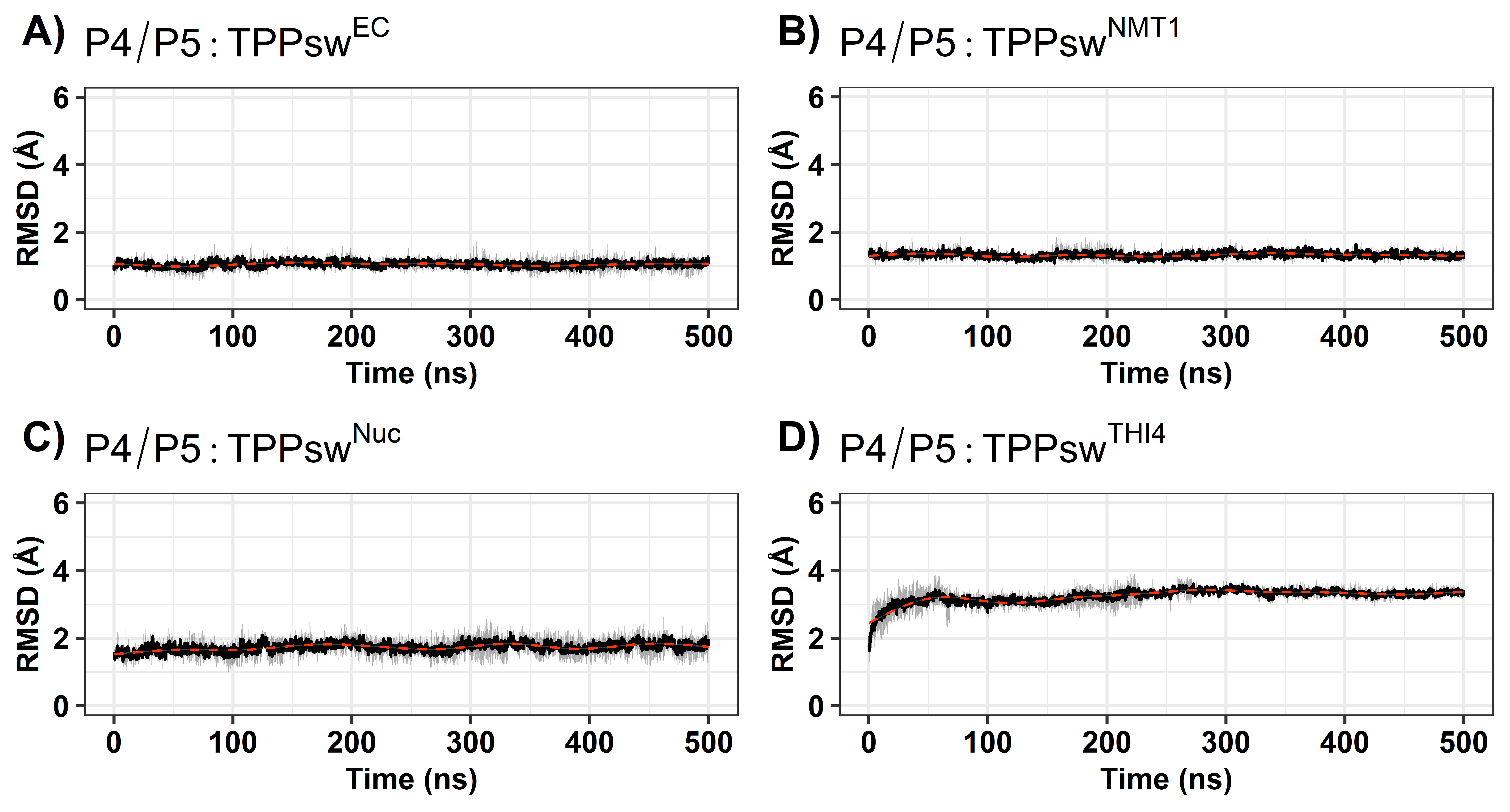


**Figure S9.** RMSD of P4 and P5 stems over 500ns. The darker line indicates the average RMSD values of the three simulations. The gray area indicates the standard deviation between the values observed in each replicate, while the red dashed line indicates the trend of the values.


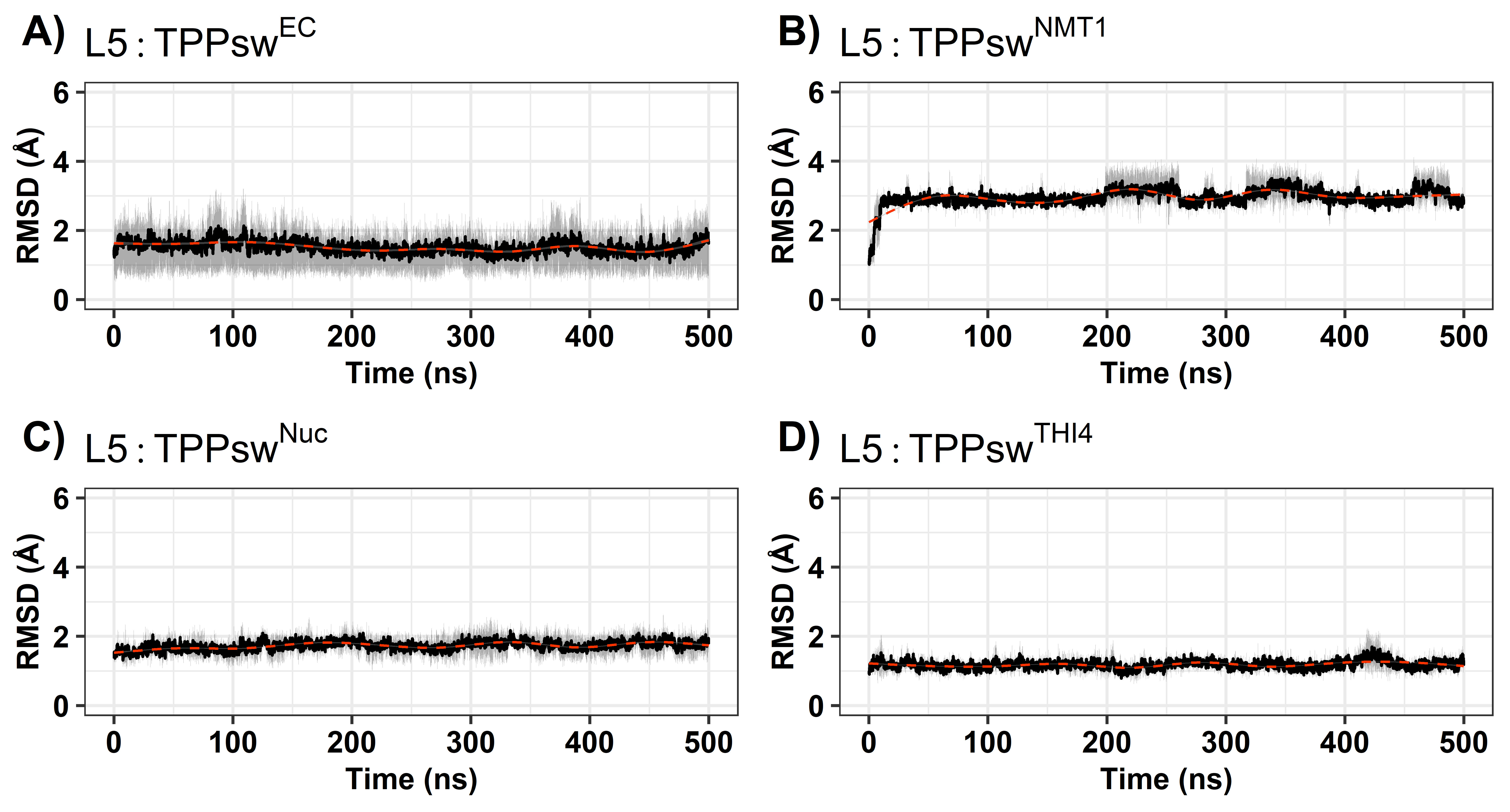


**Figure S10**. RMSD of Loop 5 over 500ns. The darker line indicates the average RMSD values of the three simulations. The gray area indicates the standard deviation between the values observed in each replicate, while the red dashed line indicates the trend of the values.


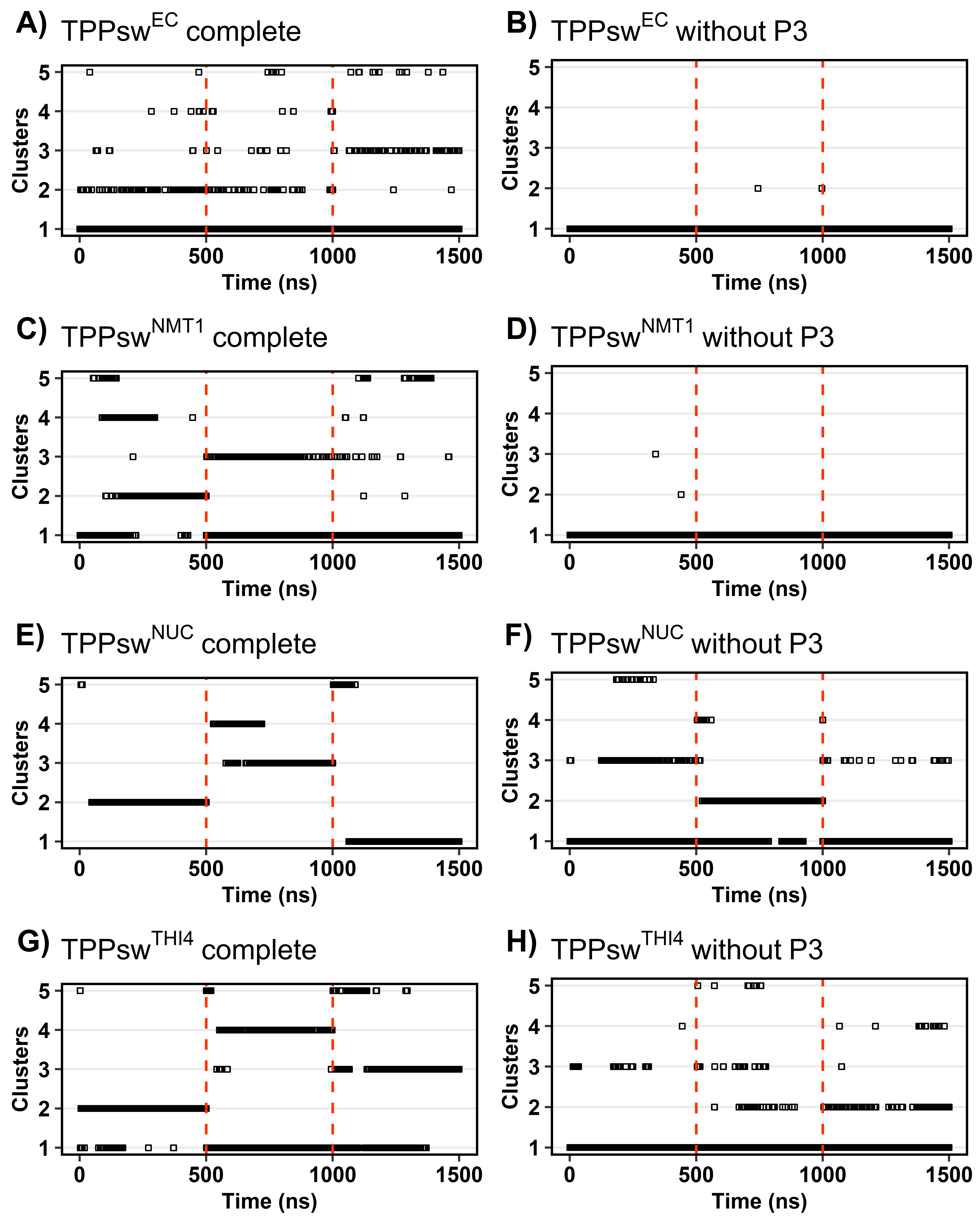


**Figure S11.** Clustering analysis of the aptamer with and without the P3 region. The replicates were concatenated, and each simulation comprises 500 ns. The dashed red lines indicate the limits of each replicate. The structures were grouped according to their RMSDs compared to the initial structure within a cutoff of 2.5 Å. Unfilled squares indicate single structures, and horizontal black bars indicate a high density of structures.

**Table S6.** RMSD between representative structures from the first 5 clusters and their respective initial structure.

|  | TPPsw^EC^ | TPPsw^NMT1^ | TPPsw^NUC^ | TPPsw^THI4^ |
| --- | --- | --- | --- | --- |
| Cluster 1 | 1.37 | 2.05 | 2.60 | 1.71 |
| Cluster 2 | 1.46 | 2.20 | 2.85 | 1.51 |
| Cluster 3 | 1.25 | 2.23 | 3.11 | 1.47 |
| Cluster 4 | 1.98 | 2.75 | 2.73 | 1.88 |
| Cluster 5 | 1.44 | 2.30 | 2.65 | 1.44 |

All values are given in Angstrons (Å).
